# Anti-Inflammatory Protein Hydrolysate Screened from Crayfish Shells Attenuates Colitis by Oxidative Stress Suppression, Inflammation Reduction and Barrier Reconstruction

**DOI:** 10.3390/foods15193389

**Published:** 2026-09-23

**Authors:** Lei Dong, Hao Wang, Yongmin Wu, Jinyang Peng, Shaohao Lai, Zexin Zhao, Yongqi Yin, Jun Cai

**Affiliations:** 1 School of Life and Health Sciences, Hubei University of Technology, No. 28 Nanli Road, Wuhan 430068, China; dll0103@163.com (L.D.); wanghao05270@163.com (H.W.); lsh990131@163.com (S.L.); zx_zhao@hbut.edu.cn (Z.Z.); 2 College of Food Science and Engineering, Yangzhou University, Yangzhou 210095, China; yqyin@yzu.edu.cn

**Keywords:** crayfish shell protein hydrolysate, colitis, anti-inflammatory activity, oxidative stress, tight junction proteins

## Abstract

Inflammatory bowel disease (IBD) is associated with intestinal inflammation and oxidative stress. Protein-rich crayfish shell (CS) by-products may provide a sustainable source of bioactive hydrolysates. This study screened five protease-derived hydrolysates and evaluated the alkaline protease-derived hydrolysate (ACSP) in lipopolysaccharide-stimulated IEC-6 cells and mice with DSS-induced colitis. ACSP scavenged DPPH and ABTS•^+^ radicals, with IC_50_ values of 0.888 ± 0.004 and 0.853 ± 0.003 mg/mL. It also enhanced superoxide dismutase and catalase activities, and inhibited NO release and albumin denaturation in vitro. In IEC-6 cells, ACSP increased viability, reduced iNOS, IL-1β, IL-6, and TNF-α expression, and increased IL-10 expression. In mice, ACSP attenuated weight loss, disease activity, colon shortening, histopathological injury, and goblet cell depletion. The high-dose group had disease activity index and histological scores of 3.60 ± 0.55 and 4.80 ± 0.40 compared with 9.40 ± 1.14 and 10.40 ± 0.80 in the model group. ACSP reduced pro-inflammatory cytokines, myeloperoxidase activity, and NO levels while restoring antioxidant enzyme activities and tight junction protein expression. Peptidomic analysis identified 516 peptide sequences, including peptides containing hydrophobic, acidic, and C-terminal basic residues. These findings support further investigation of ACSP for intestinal health applications and the value-added utilization of CS by-products.

## 1. Introduction

Changes in lifestyle, dietary habits, and environmental conditions have contributed to the increasing global prevalence of inflammatory bowel disease (IBD) [1,2,3], with more than 6.8 million cases reported worldwide according to the Global Burden of Disease Study [4]. IBD encompasses ulcerative colitis and Crohn’s disease, chronic inflammatory disorders involving interactions among genetic susceptibility, environmental factors, gut microbial dysbiosis, oxidative stress, and excessive immune activation [5,6,7,8]. Current therapeutic interventions for colitis encompass a range of pharmaceutical agents, including aminosalicylates, corticosteroids, immunomodulators, and biologics to induce and maintain remission. However, the utilization of these agents is constrained by several limitations, including adverse effects, secondary loss of response, high costs, and incomplete mucosal healing. It is evident that these limitations underscore the necessity for efficacious, multi-targeted, and safe long-term therapeutic alternatives [9].

Food-derived bioactive peptides have attracted interest for IBD management because of their reported biocompatibility, low immunogenicity, potential for oral administration, and activity across multiple disease-related pathways [10,11,12]. Peptides from animal, marine, and plant sources have been shown to possess antioxidant and anti-inflammatory activities. Reported mechanisms include free radical scavenging, enhancement of endogenous antioxidant defenses, inhibition of nuclear factor-κB (NF-κB) and mitogen-activated protein kinase (MAPK) signaling, suppression of pro-inflammatory mediators, and increased production of anti-inflammatory cytokines such as interleukin-10 (IL-10) [13,14,15,16]. Reported anti-inflammatory peptides share several sequence features, including short lengths (typically 2–20 amino acids), N-terminal hydrophobic residues that may facilitate membrane interactions, and C-terminal positively charged residues such as arginine and lysine that may favor cellular uptake or receptor binding. Residues such as leucine, serine, tyrosine, and arginine have also been associated with antioxidant and anti-inflammatory activity [17]. These features may guide the identification and design of bioactive peptides. However, gastrointestinal degradation, incompletely understood mechanisms, and the sustainability of raw materials remain barriers to clinical translation [18].

Crayfish (*Procambarus clarkii*) is widely consumed, with annual production exceeding 3 million tons. Crayfish shells (CS), which account for approximately 60–70% of total crayfish biomass, contain substantial amounts of chitin (20–30%) and protein (20–40%) with a balanced amino acid profile [19]. However, less than 10% of this material undergoes high-value utilization, contributing to resource waste and environmental burdens [20]. Proteomic analyses indicate that CS proteins include muscle-associated proteins, structural proteins, and enzymes. Many contain hydrophobic residues such as leucine and phenylalanine, as well as residues associated with antioxidant activity, including tyrosine and tryptophan [21,22]. These compositional features suggest that CS proteins may be a source of multifunctional bioactive peptides. Previous studies have reported antioxidant, antifreeze, and lipid metabolism-related activities of CS-derived products [19,23,24]. However, the ability of CS protein hydrolysates to alleviate intestinal inflammation remains insufficiently characterized. Identifying an effective enzymatic hydrolysis strategy could therefore support by-product valorization and the development of food-derived candidates for intestinal health research [25].

Bioactive peptides are commonly produced by enzymatic protein hydrolysis. Differences in protease cleavage specificity generate peptide mixtures with distinct compositions and biological activities [26,27]. Commonly used enzymes include neutral protease, alkaline protease, and papain [28]. For example, trypsin cleaves peptide bonds on the C-terminal side of arginine and lysine residues. Trypsin-derived peptides (2–4 kDa) containing hydrophobic cores have been reported to suppress inflammatory activation by blocking Toll-like receptor 4 (TLR4) [29]. Alkaline proteases can release peptides containing aromatic residues that may contribute to reactive oxygen species (ROS) scavenging and protection against oxidative damage [30]. Ma et al. [31] used papain, alkaline protease, and neutral protease to hydrolyze α-lactalbumin and reported that the neutral protease hydrolysate alleviated dextran sulfate sodium (DSS)-induced colitis in mice in association with changes in TLR4/NF-κB signaling and the gut microbiota. These findings support enzymatic hydrolysis as a strategy for producing bioactive CS protein hydrolysates. Because protease specificity influences peptide composition and activity, we compared multiple enzymes during initial screening.

To address this gap, this study aimed to prepare CS protein hydrolysates using proteases with different cleavage specificities and compare their antioxidant and anti-inflammatory activities. Hydrolysates with superior in vitro performance were further evaluated in lipopolysaccharide (LPS)-stimulated IEC-6 cells. The hydrolysate showing the strongest overall activity was subsequently examined in a DSS-induced acute colitis mouse model and to explore its mechanism. LC–MS/MS-based peptidomic analysis was also conducted to characterize the peptide composition of the selected hydrolysate. By integrating protease-dependent screening, stepwise in vitro and in vivo validation, and peptidomic characterization within a unified experimental framework, this study provides evidence for the intestinal health-related potential of CS protein hydrolysates and supports the value-added utilization of crustacean-processing by-products.

## 2. Materials and Methods

### 2.1. Materials and Reagents

CS was obtained from Shayang Chengfeng Technology Co., Ltd. (Wuhan, China). Neutral protease (D675604), alkaline protease (P769096), papain (P769096), pepsin (P6322), and trypsin (T819003) were obtained from Shanghai Macklin Biochemical Co., Ltd. (Shanghai, China). DSS (molecular weight: 36,000–50,000 Da) was purchased from MP Biomedicals (Solon, OH, USA), and LPS (ST1470) was purchased from Beyotime Biotechnology Inc. (Shanghai, China). Catalase (CAT, BS201) and superoxide dismutase (SOD, BS933) were obtained from Biosharp Biotechnology Co., Ltd. (Hefei, China). Enzyme-linked immunosorbent assay (ELISA) kits for tumor necrosis factor-α (TNF-α, SEKR-0009), interleukin-1β (IL-1β, SEKR-0002), interleukin-6 (IL-6, SEKR-0006), and IL-10 (SEKR-0010) were obtained from Beijing Solarbio Technology Co. Commercial activity assay kits for SOD, CAT, and myeloperoxidase (MPO) were purchased from Shanghai MLBIO Biotechnology Co., Ltd. (Shanghai, China). Nitric oxide (NO) and Cell Counting Kit-8 (CCK-8) assay kits were obtained from Shanghai Yuetian Biotechnology Co., Ltd. (Shanghai, China). The HiPure Total RNA Mini Kit was purchased from Magen Biotechnology Co., Ltd. Rabbit antibodies against zonula occludens-1 (ZO-1), claudin-1, occludin, and glyceraldehyde-3-phosphate dehydrogenase (GAPDH), together with a horseradish peroxidase (HRP)-conjugated anti-rabbit IgG secondary antibody, were purchased from Cell Signaling Technology, Inc. (Danvers, MA, USA).

### 2.2. Preparation of CS Protein Hydrolysates

CS was cut into small pieces, dried, mechanically ground, and passed through a 60-mesh sieve. The resulting powder was mixed with petroleum ether at a ratio of 1:10 (*w*/*v*) and stirred magnetically at room temperature for 6 h. The mixture was filtered to remove the lipid-containing solvent. The defatted powder was then mixed with 1 M HCl at a ratio of 1:10 (*w*/*v*) and decalcified for 2 h. After centrifugation at 5000× *g* for 20 min, the supernatant was discarded, and the solid residue was dried to obtain decalcified CS powder.

CS protein hydrolysates (CSPs) were prepared according to Dong et al. [27], with minor modifications. Briefly, CS powder was dispersed in distilled water at a ratio of 1:25 (*w*/*v*) and hydrolyzed under the conditions listed in Table 1. Following enzyme inactivation at 95 °C for 15 min, the hydrolysates were passed through a 0.45-μm membrane to remove insoluble material. The filtrate was collected, freeze-dried, and stored at 4 °C until use. Hydrolysates prepared using papain, alkaline protease, neutral protease, trypsin, and pepsin were designated PCSP, ACSP, NCSP, TCSP, and PECSP, respectively. Functional stability was evaluated after storage at 4 °C for 0, 7, 15, and 30 days by repeating the antioxidant and anti-inflammatory assays under identical conditions.

### 2.3. Evaluation of Antioxidant and Anti-Inflammatory Activities

#### 2.3.1. DPPH Radical Scavenging Assay

Each hydrolysate was tested at concentrations of 0.2, 0.4, 0.6, 0.8, and 1.0 mg/mL. Each sample solution (0.5 mL) was mixed with 2.5 mL of 60 μM 2,2-diphenyl-1-picrylhydrazyl (DPPH) in methanol [32]. The mixtures were vortexed for 30 s and incubated in the dark for 30 min before absorbance was measured at 517 nm. DPPH radical scavenging activity was calculated as follows. For each hydrolysate, three replicate concentration–response curves were generated by plotting DPPH radical-scavenging activity (%) against hydrolysate concentration (mg/mL). The response variable was the calculated scavenging percentage; neither the concentration nor the response data were additionally normalized or logarithmically transformed.

Scavenging activity (%) = [A_0_ − (A_1_ − A_2_)]/A_0_ × 100

where A_0_ is the absorbance of the control (0.5 mL methanol + 2.5 mL DPPH solution), A_1_ is the absorbance of the sample (0.5 mL sample solution + 2.5 mL DPPH solution), and A_2_ is the absorbance of the sample blank (0.5 mL sample solution + 2.5 mL methanol). The IC_50_ was defined as the hydrolysate concentration required to achieve 50% DPPH radical-scavenging activity.

#### 2.3.2. ABTS•^+^ Radical Scavenging Assay

ABTS•^+^ radical scavenging activity was determined as previously described [33]. Each hydrolysate was evaluated at concentrations of 0.2, 0.4, 0.6, 0.8, and 1.0 mg/mL. A stock solution of the 2,2′-azino-bis (3-ethylbenzothiazoline-6-sulfonic acid) radical cation (ABTS•^+^) was prepared by mixing 7 mM aqueous ABTS with 2.45 mM potassium persulfate at a ratio of 1:1 (*v*/*v*), followed by incubation in the dark at room temperature for 16–24 h. The stock solution was diluted with ethanol to an absorbance of 0.70 ± 0.02 at 734 nm. Sample solution (50 μL) was mixed with 1.9 mL of working solution and incubated in the dark at room temperature for 6 min. Absorbance was immediately measured at 734 nm, and radical scavenging activity was calculated as follows. The calculation method is the same as that for DPPH.

Scavenging activity (%) = (A_0_ − A_1_)/A_0_ × 100

where A_0_ is the absorbance of the blank control and A_1_ is the absorbance of the test sample. The IC_50_ was defined as the hydrolysate concentration required to achieve 50% ABTS•^+^ radical-scavenging activity.

#### 2.3.3. Ferric Reducing Antioxidant Power Assay

Ferric reducing antioxidant power (FRAP) was measured using the method of Benzie et al. [34], with modifications. The FRAP reagent was prepared by mixing 300 mM sodium acetate buffer (pH 3.6), 10 mM 2,4,6-tripyridyl-s-triazine (TPTZ) in 40 mM HCl, and 20 mM FeCl_3_ at a ratio of 10:1:1 (*v*/*v*/*v*). Freshly prepared reagent was prewarmed to 37 °C. Sample solution (0.1 mL) was mixed with 1.8 mL of FRAP reagent and 3.1 mL of ultrapure water. After incubation at 37 °C for 30 min, absorbance was measured at 593 nm. FeSO_4_ standards (100–1000 μM) were used to construct a calibration curve: y = 0.2638x + 0.0651 (R^2^ = 0.9992). Results were expressed as μmol Fe(II) equivalents per gram of dry sample.

#### 2.3.4. Hydroxyl Radical (•OH) Scavenging Assay

Salicylic acid in ethanol (1 mL, 9 mM), FeSO_4_ (1 mL, 9 mM), sample solution (1 mL, 1 mg/mL), and H_2_O_2_ (1 mL, 8.8 mM) were added sequentially, with vortexing after each addition [35]. The mixture was incubated at 37 °C for 30 min, and absorbance was measured at 510 nm. Hydroxyl radical (•OH) scavenging activity was calculated as follows:

Scavenging activity (%) = [(A_0_ − A)/A_0_] × 100

where A_0_ and A are the absorbances of the control and sample, respectively.

#### 2.3.5. Superoxide Anion Radical (O_2_•^−^) Scavenging Assay

Superoxide anion radical (O_2_•^−^) scavenging activity was evaluated by monitoring catechol autoxidation. Catechol solution (0.5 mL, 10 mM) was mixed with 4.5 mL of Tris-HCl buffer (50 mM, pH 8.2), followed by 1 mL of sample solution (1 mg/mL). The mixture was incubated at 25 °C for 5 min, and the reaction was stopped by adding 1 mL of 8% HCl. Absorbance was measured at 320 nm within 5 min of reaction termination. Scavenging activity was calculated as follows:

Scavenging activity (%) = [1 − (ΔA_s_ − ΔA_b_)/ΔA_0_] × 100

where ΔA_s_, ΔA_b_, and ΔA_0_ are the changes in absorbance from 0 to 5 min for the sample, sample blank, and water control, respectively.

#### 2.3.6. Cell-Free Assay of SNP-Derived NO Release Inhibition

Sample solution (40 μL) was mixed with 260 μL of phosphate-buffered saline (PBS; 0.1 M, pH 7.4) and 100 μL of sodium nitroprusside (SNP; 100 mM), then incubated under illumination at 37 °C for 2 h. An aliquot (100 μL) was reacted with Griess reagent, prepared by mixing equal volumes of 1% (*w*/*v*) sulfanilamide in 5% H_3_PO_4_ and 0.1% (*w*/*v*) NEDD (Shanghai Yuetian Biotechnology Co., Ltd., Shanghai, China). After incubation in the dark for 10 min, absorbance was measured at 540 nm. For the blank control, ultrapure water was used instead of the sample solution [35]. Inhibition of SNP-derived NO release was calculated as follows:

NO release inhibition (%) = (A_blank_ − A_sample_)/A_blank_ × 100

#### 2.3.7. Albumin Denaturation Assay

Inhibition of albumin denaturation was assessed as described by Hu et al. [36]. Sample solution (50 μL) was mixed with 450 μL of bovine serum albumin (BSA; 1% *w*/*v* in 0.01 M PBS, pH 7.4). The mixtures were incubated at 37 °C for 15 min and then heated at 90 °C for 20 min to induce denaturation. Turbidity was measured at 660 nm and 25 °C using a microplate reader. For the blank control, ultrapure water was used instead of the sample solution. Inhibition of denaturation was calculated as follows:

Denaturation inhibition (%) = (A_blank_ − A_sample_)/A_blank_ × 100

### 2.4. Determination of SOD and CAT Activities

The effects of the five hydrolysates on SOD and CAT activities were evaluated using the corresponding commercial activity assay kits. SOD and CAT activities in colon homogenates and serum were measured using kits from Shanghai MLBIO Biotechnology Co., Ltd. (Shanghai, China), according to the manufacturer’s instructions. Absorbance was measured at 450 nm for SOD and 240 nm for CAT.

### 2.5. Cell Culture and Viability Assays

IEC-6 cells (BeNa Culture Collection; BNCC100548) were maintained in Dulbecco’s modified Eagle medium (DMEM) supplemented with 10% fetal bovine serum (FBS), 100 U/mL penicillin, 100 μg/mL streptomycin, and 0.1 IU/mL insulin at 37 °C in an atmosphere containing 5% CO_2_.

For the viability time-course assay, IEC-6 cells (1 × 10^5^ cells/mL) in 96-well plates were treated with ACSP or NCSP (0.1–2.0 mg/mL) for up to 48 h. Viability was assessed using a CCK-8 assay kit (Shanghai Yuetian Biotechnology Co., Ltd., Shanghai, China) at 0, 12, 24, 36, and 48 h. At each time point, CCK-8 reagent was added at 10% (*v*/*v*), and the cells were incubated for a further 2 h before absorbance was measured.

For the inflammation experiments, IEC-6 cells were seeded in 12-well plates at 3 × 10^5^ cells/well and cultured for 12 h. Cells were stimulated with LPS (0.1 mg/mL) for 4 h and then treated with CSPs (0.025–0.5 mg/mL) for 24 h. Cells and culture supernatants were collected for quantitative polymerase chain reaction (qPCR) and ELISA analyses, respectively.

### 2.6. Animals and Experimental Design

Thirty male C57BL/6 mice (6 weeks old; 20 ± 1 g) were obtained from Luobin Life Technology Co., Ltd. (Wuhan, China). Mice were housed at 24 ± 1 °C under a 12-h light/dark cycle with free access to food and water. After 3 days of acclimation, eligible animals were assigned to six groups (*n* = 5 per group) using a random-number procedure. Group size was determined before the experiment by an a priori power analysis; the calculation assumptions are provided in the Supplementary Methods. No animals or data points were excluded from the final analysis. The groups were: control (Control); DSS model (Model); mesalazine positive control (Positive; 200 mg/kg body weight); and low-, medium-, and high-dose ACSP groups (LACSP, MACSP, and HACSP; 100, 200, and 400 mg/kg body weight, respectively). All groups except Control received 3% DSS (*w*/*v*) in drinking water ad libitum for 7 days. DSS was then withdrawn, and all groups received normal drinking water during the subsequent 7-day treatment period. During treatment, the Model group received PBS, the Positive group received mesalazine in PBS, and the ACSP groups received ACSP in PBS at their assigned doses. Treatments were administered by oral gavage once daily for 7 consecutive days at 1 mL per mouse. Control mice received normal drinking water without gavage. Body weight, food consumption, general clinical condition, and fecal characteristics were assessed once daily at a fixed time by personnel blinded to group allocation (Further details are provided in the Supplementary Materials). After the final treatment, mice were fasted for 24 h, and orbital blood was collected under anesthesia. Mice were then euthanized by cervical dislocation. The colon, thymus, and spleen were excised, rinsed in ice-cold saline, and measured. Distal colon segments were fixed in 4% paraformaldehyde for histology, middle segments were snap-frozen in liquid nitrogen for qPCR and Western blotting, and proximal segments were stored at −80 °C for ELISA and oxidative stress assays. Analyses were performed on coded samples by investigators blinded to group allocation until data analysis was complete. The experimental timeline is shown in Figure 3A.

### 2.7. Assessment of Colitis Severity

Colitis severity was assessed using the criteria described by Wan et al. [37], including rectal bleeding, body weight loss, and stool consistency (Table S1), by personnel blinded to group allocation. Body weight loss was calculated as a percentage of the baseline weight recorded before the first DSS exposure. Stool consistency was assessed by direct visual inspection of freshly voided feces, based on pellet formation and water content. Rectal bleeding was evaluated by visual inspection of freshly voided feces and the perianal region for grossly visible blood. Each component was scored from 0 to 4, and the disease activity index (DAI) was calculated as the sum of the three scores:

DAI = body weight loss score + stool consistency score + rectal bleeding score

### 2.8. Immune Organ Index

The thymus and spleen were blotted dry and weighed. The organ index was calculated as follows:

Organ index = organ weight/body weight

### 2.9. Histopathology

Colon tissues fixed in 4% paraformaldehyde were embedded in paraffin, sectioned at 5 μm, and stained with hematoxylin and eosin (H&E) [38]. Histological injury was scored according to the depth of inflammatory infiltration, crypt damage, and extent of the affected area (Table S2). Goblet cells were identified by Alcian blue–periodic acid–Schiff (AB-PAS) staining. Images were acquired using a microscope (Olympus BH2, Tokyo, Japan).

### 2.10. Gene Expression Analysis

Total RNA was extracted from IEC-6 cells and colon tissues using the HiPure Total RNA Mini Kit (Magen Biotechnology Co., Ltd., Guangzhou, China) according to the manufacturer’s instructions. The purity of extracted RNA was determined using a NanoDrop (Thermo Scientific, Waltham, WA, USA). Samples with absorbance ratios ranging from 1.8 to 2.1 were retained for subsequent experiments. Complementary DNA (cDNA) was synthesized using a reverse transcription kit (Thermo Scientific, USA). Quantitative PCR was performed using Power SYBR Green PCR Master Mix (Agbio, Changsha, China) on a QuantStudio 6 Flex Real-Time PCR System (Thermo Scientific, USA) to measure the relative mRNA expression of inducible nitric oxide synthase (iNOS), TNF-α, IL-1β, IL-6, IL-10, ZO-1, claudin-1, and occludin. Relative expression was calculated using the 2^−ΔΔCt^ method, with GAPDH as the endogenous reference gene, and the results were normalized. General clinical condition primer sequences are listed in Table S3.

### 2.11. Enzyme-Linked Immunosorbent Assay

TNF-α, IL-1β, IL-6, and IL-10 concentrations in IEC-6 culture supernatants and colon homogenates were measured using the corresponding ELISA kits (Beijing Solarbio Science & Technology Co., Ltd., Beijing, China) according to the manufacturer’s instructions. MPO activity in colon homogenates and serum was measured separately using a commercial activity assay kit (Shanghai MLBIO Biotechnology Co., Ltd., Shanghai, China).

### 2.12. Immunofluorescence

Paraffin-embedded colon tissues were sectioned at 5 μm, deparaffinized, and rehydrated through a graded ethanol series. Heat-induced antigen retrieval was performed in 10 mM sodium citrate buffer (pH 6.0) under pressure for 3 min. After cooling to room temperature, sections were permeabilized and blocked with 3% BSA for 1 h at 37 °C. Sections were incubated overnight at 4 °C with rabbit primary antibodies against ZO-1, claudin-1, or occludin. After three washes with PBS, sections were incubated with a fluorescein isothiocyanate (FITC)-conjugated goat anti-rabbit IgG secondary antibody for 1 h at room temperature in the dark. Nuclei were counterstained with 4′,6-diamidino-2-phenylindole (DAPI) for 30 min [39]. Images were acquired using a microscope (Zeiss LSM 980 Airyscan 2, Aubochingen, Germany), and fluorescence intensity was quantified using ImageJ version 1.8.0.

### 2.13. Western Blotting

Colon tissues were homogenized in radioimmunoprecipitation assay (RIPA) lysis buffer on ice for 30 min and centrifuged at 10,000× *g* for 10 min at 4 °C. The supernatants were collected, and nuclear proteins were obtained using a nuclear and cytoplasmic protein extraction kit (Thermo Fisher, Waltham, MA, USA). Protein concentrations were measured using a bicinchoninic acid (BCA) assay. Equal amounts of protein were separated by 12.5% sodium dodecyl sulfate–polyacrylamide gel electrophoresis (SDS-PAGE) and transferred to polyvinylidene fluoride (PVDF) membranes (Merck Millipore, Darmstadt, Germany) using a semi-dry transfer system (Trans-Blot SD, Bio-Rad, Heracles, CA, USA) at 18 V for 20 min. Membranes were blocked with 5% (*w*/*v*) nonfat dry milk in Tris-buffered saline containing 0.1% Tween-20 (TBST) for 1 h at room temperature and incubated overnight at 4 °C with antibodies against ZO-1 (Cat. No. 13663; 1:1000), claudin-1 (Cat. No. 13255; 1:1000), occludin (Cat. No. 91131; 1:1000), or GAPDH (clone 14C10; Cat. No. 2118; 1:1000) (Cell Signaling Technology, Danvers, MA, USA). After three 10-min washes with TBST, membranes were incubated with HRP-conjugated anti-rabbit IgG (Cat. No. 7074; 1:2000) for 1 h at room temperature. Bands were visualized using an enhanced chemiluminescence substrate. Band intensities were quantified after background subtraction using ImageJ version 1.8.0. ZO-1, claudin-1, and occludin signals were normalized to the corresponding GAPDH signal from the same sample and expressed relative to the control group. Three independent colon samples per group, each from a different mouse, were analyzed (n = 3 biological replicates). Technical measurements were not treated as independent biological replicates.

### 2.14. Peptidomic Characterization of ACSP by LC–MS/MS

Peptides were analyzed by LC–MS/MS using an EASY-nLC 1000 system coupled to a QE mass spectrometer (Thermo Scientific, USA). ACSP was dissolved in 0.1% formic acid and centrifuged at 14,000× *g* for 20 min at 4 °C. An aliquot of the supernatant (3 μL, 1 mg/mL) was injected onto a PepMap RSLC C18 column (Thermo Scientific, USA) at a flow rate of 250 nL/min. Mobile phases A and B were ultrapure water containing 0.1% formic acid and acetonitrile, respectively. The elution gradient is provided in Table S4.

Raw LC–MS/MS data were searched against the UniProtKB *Procambarus clarkii* protein database using PEAKS Studio 11.5, build 20230821 (Bioinformatics Solutions Inc., Waterloo, ON, Canada). False discovery rates (FDRs) were estimated using a target–decoy strategy. Enzyme specificity was set to “None” and digestion mode to “unspecific”; the maximum missed-cleavage setting was three, and allowed peptide lengths ranged from 3 to 45 amino acids. Precursor and fragment mass tolerances were 10 ppm and 0.02 Da, respectively. Protein N-terminal acetylation (+42.01 Da) and histidine/tryptophan oxidation (+15.99 Da) were specified as variable modifications, with a maximum of two modifications per peptide. Identifications were filtered at a PEAKS −10lgP score of ≥37.04, yielding FDRs of 1.0% at the peptide-spectrum match level and 1.4% at the peptide-sequence level. Quality control included examination of the target–decoy FDR curve, −10lgP score distribution, score-versus-precursor-mass-error plot, and precursor mass error distribution (Figure S2). Representative high-confidence sequence assignments were supported by annotated MS/MS spectra with matched b- and y-type fragment ions (Figure S3). Search parameters and peptide identification results are provided in Tables S5 and S6, respectively.

### 2.15. Statistical Analysis

Data were analyzed using GraphPad Prism 8 (GraphPad Software Inc., San Diego, CA, USA) and are presented as the mean ± standard deviation (SD). Experiments included at least three biologically independent samples or independent experimental repeats, as specified in the figure legends. Before parametric testing, the normality of model residuals and the homogeneity of variance among groups were assessed using the Shapiro–Wilk and Levene tests, respectively. Longitudinal DAI and relative body weight data were analyzed using two-way mixed-design repeated-measures analysis of variance (ANOVA), with treatment group as the between-subject factor, time as the within-subject factor, and mouse as the repeated-measures subject. When the group × time interaction was significant, prespecified comparisons with the DSS model group at the end of treatment were performed using Dunnett’s multiple-comparisons test. Endpoints measured once at the end of the experiment were analyzed using one-way ANOVA followed by Tukey’s multiple-comparisons test when the assumptions of normality and homogeneity of variance were satisfied. All tests were two-sided, and *p* < 0.05 was considered statistically significant. Significance levels are defined in the figure legends.

## 3. Results

### 3.1. Evaluation of the In Vitro Oxidative Stress Capacity and Anti-Inflammatory Activity of Different CSPs

To identify hydrolysates with strong antioxidant potential, we systematically compared the antioxidant and anti-inflammatory activities of five protease-derived CSPs. ACSP and NCSP showed the strongest overall antioxidant activity (Table 2). Their DPPH IC_50_ values were 0.888 ± 0.004 and 1.094 ± 0.041 mg/mL, respectively, and their ABTS•^+^ IC_50_ values were 0.853 ± 0.003 and 0.794 ± 0.081 mg/mL. NCSP also had the highest FRAP value (47.637 ± 4.728 μmol Fe(II)/g), indicating the greatest reducing capacity. ACSP showed the highest hydroxyl radical scavenging activity (29.525 ± 0.499%). ACSP and NCSP also exhibited superoxide anion radical scavenging activities of 47.310 ± 1.351% and 38.225 ± 1.025%, respectively. Conversely, TCSP and PECSP consistently showed the weakest antioxidant performance across all assays. In the enzyme assays (Figure 1A), ACSP produced the largest increases in SOD and CAT activities, with NCSP showing comparable effects. The increases with ACSP were approximately 25% for SOD and greater than 18% for CAT, both significantly higher than those with PCSP and PECSP (*p* < 0.05), indicating that ACSP and NCSP could enhance antioxidant defense in vitro.

ACSP and NCSP also showed the strongest activities in the cell-free NO release and albumin denaturation assays (Figure 1B). ACSP inhibited SNP-derived NO release by more than 20%, followed by NCSP. ACSP also produced the greatest inhibition of heat-induced albumin denaturation (12.21%). As a key pro-inflammatory mediator, NO amplifies macrophage activation and oxidative stress. Together, these results indicated that ACSP and NCSP had the strongest combined antioxidant activities and anti-inflammatory potential among the five hydrolysates, supporting their selection for cellular evaluation.

### 3.2. Effects of ACSP and NCSP on LPS-Induced Inflammatory Responses in IEC-6 Cells

ACSP and NCSP were further evaluated in IEC-6 cells. ACSP increased cell viability over time, with the largest increase at 36 h observed at 0.05 mg/mL (Figure 2A). NCSP also increased viability, with the largest effect at 36 h observed at 0.25 mg/mL (Figure 2B). Subsequently, we analyzed key inflammatory mediators to quantify anti-inflammatory efficacy. LPS stimulation significantly upregulated pro-inflammatory genes (iNOS, IL-1β, IL-6, TNF-α) while downregulating anti-inflammatory IL-10 (Figure 2C–G). Both hydrolysates attenuated these changes, with ACSP generally showing stronger effects. ELISA measurements of culture supernatants showed corresponding decreases in IL-1β, IL-6, and TNF-α and an increase in IL-10 (Figure 2H–K). Overall, ACSP exhibits anti-inflammatory superiority, highlighting its potential as a functional food-derived hydrolysate for mitigating intestinal inflammation.

### 3.3. Effects of ACSP on Disease Manifestations in DSS-Induced Colitis

To evaluate ACSP’s protective effects against IBD, we established a colitis model by administering 3% DSS to mice. This model recapitulated characteristic colitis symptoms, including progressive weight loss, diarrhea, and bloody stools. After 7 days of DSS exposure, body weight was significantly lower in the Model group than in the Control group (*p* < 0.01). ACSP attenuated weight loss in a dose-related manner, with the greatest effect in the HACSP group (Figure 3C). Two-way repeated-measures ANOVA identified significant effects of treatment group, time, and their interaction on DAI and relative body weight (all *p* < 0.0001). DAI increased during DSS induction and remained elevated in the Model group after DSS withdrawal. Mesalazine and ACSP progressively reduced DAI and improved weight recovery during the 7-day treatment period. On day 14, DAI values were 9.40 ± 1.14 in the Model group and 5.60 ± 0.55, 4.20 ± 0.84, and 3.60 ± 0.55 in the LACSP, MACSP, and HACSP groups, respectively (all adjusted *p* < 0.0001 vs. Model). Relative body weight was also significantly higher in all ACSP groups than in the Model group. ACSP reduced DSS-associated splenomegaly and the spleen-to-body weight ratio in a dose-related manner (Figure 3E,G). DSS shortened the colon to 5.38 ± 0.27 cm, compared with 6.6 ± 0.15 cm in controls (*p* < 0.01). Colon lengths were 5.84 ± 0.09, 6.14 ± 0.3, and 6.38 ± 0.13 cm in the LACSP, MACSP, and HACSP groups, respectively, and 6.42 ± 0.3 cm in the Positive group (*p* < 0.05 vs. Model; Figure 3D,F). Therefore, ACSP dose-dependently alleviated multiple disease-associated changes, including body-weight loss, DAI elevation, splenomegaly, and colon shortening, supporting its protective effects in the DSS-induced colitis model.

**Figure 3:**
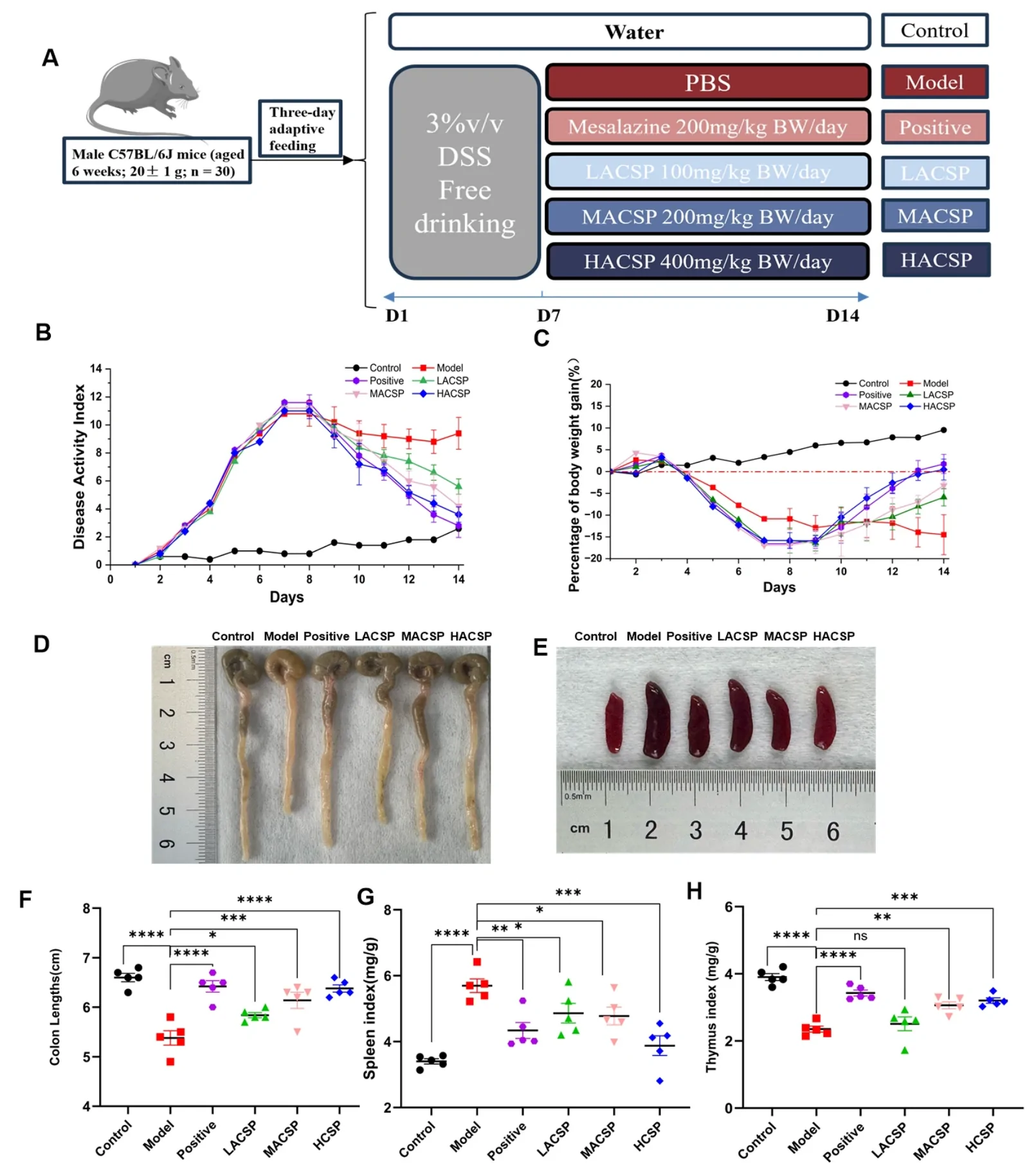
Experimental design and disease-related outcomes in mice with DSS-induced colitis treated with ACSP. (**A**) Experimental timeline. (**B**) DAI. (**C**) Percentage change in body weight. (**D**,**E**) Representative macroscopic images of the colon and spleen, respectively. (**F**) Colon length. (**G**) Spleen index. (**H**) Thymus index. Data are presented as the mean ± SD (*n* = 5). DAI (**B**) and body weight (**C**) were analyzed using two-way repeated-measures ANOVA with the Geisser–Greenhouse correction. F-H were analyzed separately using one-way ANOVA followed by Tukey’s multiple-comparisons test. ns, not significant (*p* > 0.05); * *p* < 0.05, ** *p* < 0.01, *** *p* < 0.001, **** *p* < 0.0001.

### 3.4. Effects of ACSP on Colonic Histopathology in Mice with DSS-Induced Colitis

Histopathological evaluation (Figure 4) provides compelling evidence that ACSP significantly ameliorates the colonic tissue damage characteristic of DSS-induced colitis in mice (Figure 4). H&E-stained sections from the Model group showed mucosal ulceration, crypt distortion, dense inflammatory infiltrates, and epithelial erosion (Figure 4A). ACSP improved crypt architecture and reduced ulceration and inflammatory infiltration in a dose-related manner. AB-PAS staining showed recovery of goblet cells (Figure 4B). Goblet cell counts decreased from 26.0 ± 2.77 cells/crypt in controls to 3.3 ± 1.73 in the Model group (*p* < 0.0001). HACSP increased the count to 16.4 ± 1.69 cells/crypt (*p* < 0.0001 vs. Model), compared with 19.0 ± 2.23 in the Positive group (Figure 4C). Histological scores increased from 0.5 ± 0.2 in controls to 10.4 ± 0.8 in the Model group (*p* < 0.0001) and decreased to 4.8 ± 0.4 with HACSP (*p* < 0.0001 vs. Model; Figure 4D). These in vivo protective effects align with ACSP’s established antioxidant/anti-inflammatory activities, suggesting that it has attenuated colonic mucosal injury and preserved goblet-cell-associated histological features.

### 3.5. Effects of ACSP on Inflammatory and Oxidative Stress Markers in Mice with DSS-Induced Colitis

To delineate ACSP’s anti-inflammatory mechanisms in vivo, we quantified key cytokines and oxidative stress markers in colonic tissues from DSS-colitis mice. ACSP exerted dual immunomodulation by coordinately suppressing pro-inflammatory mediators while enhancing anti-inflammatory responses. Compared with the Model group, ACSP reduced IL-1β, IL-6, and TNF-α mRNA expression by 38–82% (Figure 5A,B,D; *p* < 0.001) and their protein concentrations by 35–87% (Figure 5E,F,H; *p* < 0.0001), with dose-related effects. In the HACSP group, IL-10 mRNA and protein levels were 5.8 and 1.67 times those in the Model group, respectively (Figure 5C,G; *p* < 0.01). DSS reduced SOD and CAT activities and increased MPO activity, whereas ACSP restored antioxidant enzyme activities and reduced MPO activity (Figure 5I–K; *p* < 0.001 or *p* < 0.0001). These changes were consistent with improved antioxidant defenses and reduced neutrophil-associated inflammation. DSS also increased NO levels, and HACSP reduced colonic NO levels by more than 80% (Figure 5L; *p* < 0.0001), consistent with reduced nitrosative stress. In summary, ACSP mitigates DSS-induced colonic inflammation by suppressing pro-inflammatory cytokine expression, enhancing anti-inflammatory mediator production, and restoring antioxidant defense. These data complement the histological and goblet cell protective effects described in Figure 4, supporting the multifunctional role of ACSP in maintaining intestinal homeostasis during colitis.

### 3.6. Effects of ACSP on Colonic Tight Junction Markers in Mice with DSS-Induced Colitis

We evaluated ACSP’s effects on tight junction (TJ) components, critical determinants of intestinal barrier function, in DSS-colitis mice. DSS challenge severely disrupted TJ architecture, as evidenced by reductions in ZO-1, Claudin-1, and Occludin mRNA (Figure 6A–C, *p* < 0.001). ACSP restored their expression in a dose-related manner; ZO-1 mRNA and protein levels increased by more than 70% relative to the Model group (*p* < 0.01), with similar trends for claudin-1 and occludin. Immunofluorescence analysis showed reduced fluorescence intensity and a more fragmented distribution of these proteins after DSS exposure (Figure 6D–F,H). ACSP increased their abundance and improved their distribution along the epithelial membrane. ZO-1 fluorescence intensity in the ACSP-treated group was 8.9 times that in the Model group (*p* < 0.001), with similar trends for claudin-1 and occludin. Western blotting also showed increased TJ protein abundance after ACSP treatment (Figure 6G). Collectively, these data suggest that ACSP effectively improved intestinal barrier integrity by upregulating TJ proteins. The preservation of ZO-1, claudin-1, and occludin supports the hypothesis that ACSP alleviates colitis not only by suppressing oxidative stress and inflammation but also by maintaining mucosal homeostasis.

### 3.7. Peptidomic Characterization of ACSP

LC–MS/MS analysis identified 516 peptide sequences in ACSP, confirming its composition as a complex peptide-containing hydrolysate. The identified peptides ranged from 3 to 26 amino acids in length. By sequence count, 54.37% had molecular masses of 500–1000 Da and 41.94% had molecular masses of 1000–3000 Da (Figure 7). The ten highest-confidence sequence assignments, ranked by the PEAKS −10lgP score, are presented in Table 3. The four highest-scoring sequences were GESDDDDVVAK, SDDDDVVAK, SDLGHGIVLPPL, and YDESGPGIVH. Functional annotation indicated that numerous peptides were derived from crustacean muscle proteins (e.g., actin, myosin light chain) and cuticular proteins, which contain known immunomodulatory bioactive motifs. This protein origin suggests ACSP functions not merely as a structural hydrolysate but as a reservoir of functional sequences that may promote mucosal repair and immune homeostasis. Hydrophobic, aromatic, and acidic residues in these sequences provide a basis for investigating potential relationships between peptide composition and the antioxidant, anti-inflammatory, and TJ-associated effects observed in vivo (Figure 5 and Figure 6).

## 4. Discussion

This study compared five protease-derived CSPs and evaluated ACSP, the hydrolysate with the strongest overall activity, using chemical assays, LPS-stimulated IEC-6 cells, and a DSS-induced acute colitis mouse model. ACSP exhibited antioxidant and anti-inflammatory activities in vitro, attenuated LPS-induced inflammatory responses in IEC-6 cells, and improved disease-related, histological, biochemical, and TJ-associated outcomes in mice. These effects were accompanied by lower pro-inflammatory cytokine levels, restored antioxidant enzyme activities, and increased expression of epithelial TJ markers, supporting further investigation of ACSP as a food-derived bioactive hydrolysate.

Screening revealed protease-dependent differences in bioactivity, with ACSP and NCSP showing the strongest overall effects under the tested conditions. Differences in peptide composition may contribute to these activities. Residues such as histidine, leucine, and cysteine may influence antioxidant activity through distinct structural and chemical properties [40,41,42]. The low DPPH and ABTS•^+^ IC_50_ values, high reducing capacity, and effects on SOD and CAT activities were consistent with antioxidant potential. These findings support the hypothesis that protease specificity influences the release of bioactive peptides. Inhibition of SNP-derived NO release and heat-induced albumin denaturation provided additional evidence of activity in cell-free screening assays. These results support further investigation of protein stabilization and redox-related mechanisms but do not directly establish modulation of macrophage-derived mediators.

The biological effects of ACSP may reflect several complementary processes. One possible mechanism underlying the antioxidant activity of ACSP is that aromatic and other redox-active amino acid (Tyr, Phe, Trp) residues donate electrons or hydrogen atoms, thereby limiting ROS accumulation. Reduced oxidative stress may subsequently attenuate redox-sensitive MAPK and NF-κB signaling and create conditions favorable for the recovery of TJ proteins (ZO-1, claudin-1, and occludin) and goblet cells [43]. The observed reductions in NO levels and MPO activity are also consistent with reduced nitrosative stress and neutrophil-associated inflammation. These changes may help preserve epithelial structure, given the reported contribution of excessive nitrosative stress to crypt loss and mucosal atrophy [44].

Among the 516 LC–MS/MS-identified peptide sequences, 56.51% had molecular masses of ≤1 kDa. The hydrolysate contained short peptides with acidic and aromatic residues, as well as GESDDDDVVAK, one of the highest-confidence sequence assignments. Recurring sequence features may help explain the hydrolysate’s bioactivity [45]. C-terminal residues such as leucine or arginine may influence membrane interactions and peptide uptake [46]. Acidic residues such as aspartate and glutamate may contribute to metal chelation and limit Fenton-type radical formation, whereas tyrosine and tryptophan may participate in radical scavenging through hydrogen atom or electron donation. Hydrophobic and proline-rich segments may influence membrane association and proteolytic stability [19]. Peptides assigned to myosin light chain, actin, hemocyanin, and calcification-associated matrix proteins therefore provide candidates for investigating structure–activity relationships. Their individual contributions to ACSP’s antioxidant and anti-inflammatory effects remain to be established.

A possible signaling model is that ACSP lowers ROS and reactive nitrogen species (RNS) levels, thereby attenuating redox-sensitive MAPK and IκB kinase (IKK)/NF-κB signaling. This model is consistent with reduced NO levels and MPO activity, lower expression of pro-inflammatory mediators such as iNOS, IL-1β, IL-6, and TNF-α, and increased IL-10 expression. Another possibility involves AMP-activated protein kinase (AMPK), which may interact with NF-κB signaling through IKK and influence myosin light chain kinase (MLCK)-dependent myosin light chain (MLC) phosphorylation. Such effects could limit actomyosin contraction and help preserve the abundance and localization of ZO-1, claudin-1, and occludin. Reduced oxidative and nitrosative modification of TJ proteins may also contribute to epithelial protection [47]. These pathways provide hypotheses for the coordinated inflammatory and TJ-associated changes observed in this study.

In IEC-6 cells, ACSP increased viability and attenuated LPS-induced changes in inflammatory markers. The small size, amphiphilic properties, and aromatic residues of some constituent peptides may influence interactions with cellular targets, including IKKβ and the MAPKs p38 and c-Jun N-terminal kinase (JNK), as well as nuclear factor erythroid 2-related factor 2 (Nrf2)–Kelch-like ECH-associated protein 1 (Keap1) signaling [48]. These proposed interactions require direct binding studies and targeted pathway perturbation experiments.

In mice with DSS-induced colitis, ACSP alleviated weight loss, elevated DAI, colon shortening, and splenomegaly in a dose-related manner. Histological improvements included better-preserved colonic architecture, reduced inflammatory infiltration, and recovery of goblet cells. These findings support the relevance of preserving epithelial and mucus-associated features during colitis [49]. ACSP reduced IL-1β, IL-6, and TNF-α expression and increased IL-10 expression at both the mRNA and protein levels. The accompanying reductions in NO levels and MPO activity are compatible with attenuation of inflammatory processes involving NF-κB [50], while higher SOD and CAT activities indicate improved antioxidant defenses [51]. Simultaneously, ACSP also increased ZO-1, claudin-1, and occludin expression and improved their epithelial distribution. TJ proteins and the mucus layer contribute to restricting the passage of pathogens and bacterial products across the intestinal epithelium [38,52]. Additionally, goblet cells are essential for mucin secretion and barrier function, with studies suggesting that their depletion exacerbates colitis [48]. The coordinated changes in TJ markers, goblet cells, and histological injury support an association between ACSP treatment and preservation of epithelial structure. AMPK–MLCK–MLC signaling is one possible explanation for these changes, but its involvement remains to be tested.

The findings can be considered alongside studies of other aquatic protein hydrolysates. Ambigaipalan et al. [21] reported antioxidant activities in hydrolysates derived from shrimp shell processing discards, consistent with the reducing and radical scavenging activities observed here. Chi et al. [53] examined the relationship between amino acid composition, peptide profiles, and antioxidant activity in skipjack tuna protein hydrolysates. Differences in protein substrates and hydrolysis conditions may therefore contribute to variation among studies. Yu et al. [54] reported that sea cucumber peptides alleviated DSS-induced colitis in association with changes in the intestinal barrier and immune responses. The dose-related recovery of goblet cells in the present study provides a basis for further dose selection in studies of CS hydrolysates. Jiang et al. [55] investigated the antioxidant and antibacterial activities of Maillard-modified crab shell peptides, illustrating another approach to modifying shell-derived peptide activity. Together, these studies support further investigation of aquatic by-products as sources of bioactive hydrolysates, while the present work links protease screening with cellular and animal evaluation of ACSP.

This study identifies ACSP as a bioactive CS protein hydrolysate and supports the value-added utilization of freshwater crustacean shell by-products. Its antioxidant and anti-inflammatory activities and effects on epithelial structure warrant further preclinical evaluation. Compared with extensively studied milk-, fish-, and soy-derived peptides, crustacean shell hydrolysates remain less well characterized. The present findings expand the evidence for their potential use in intestinal health research.

Several limitations should be considered. ACSP is a complex protein hydrolysate, and the contributions of individual peptides to its activity remain unresolved. Intestinal permeability, gastrointestinal stability, epithelial transport, systemic exposure, and pharmacokinetics were not directly assessed. The proposed signaling pathways require targeted validation. Comprehensive safety, allergenicity, and clinical evaluations were also outside the scope of this study, and the in vivo findings are limited to a short-term DSS-induced acute colitis model in mice. Further work should combine bioactivity-guided fractionation, direct permeability and transport assays, targeted peptide detection, and systematic safety assessment.

## 5. Conclusions

ACSP, an alkaline protease-derived CS protein hydrolysate, attenuated disease-associated changes in mice with DSS-induced acute colitis and improved inflammatory, oxidative stress, histological, and TJ-associated markers. Its peptide composition, shaped by the source proteins and hydrolysis conditions, may contribute to these effects. The findings support further preclinical investigation of CS protein hydrolysates for intestinal inflammation and the value-added utilization of crustacean shell by-products.

## Figures and Tables

**Figure 1:**
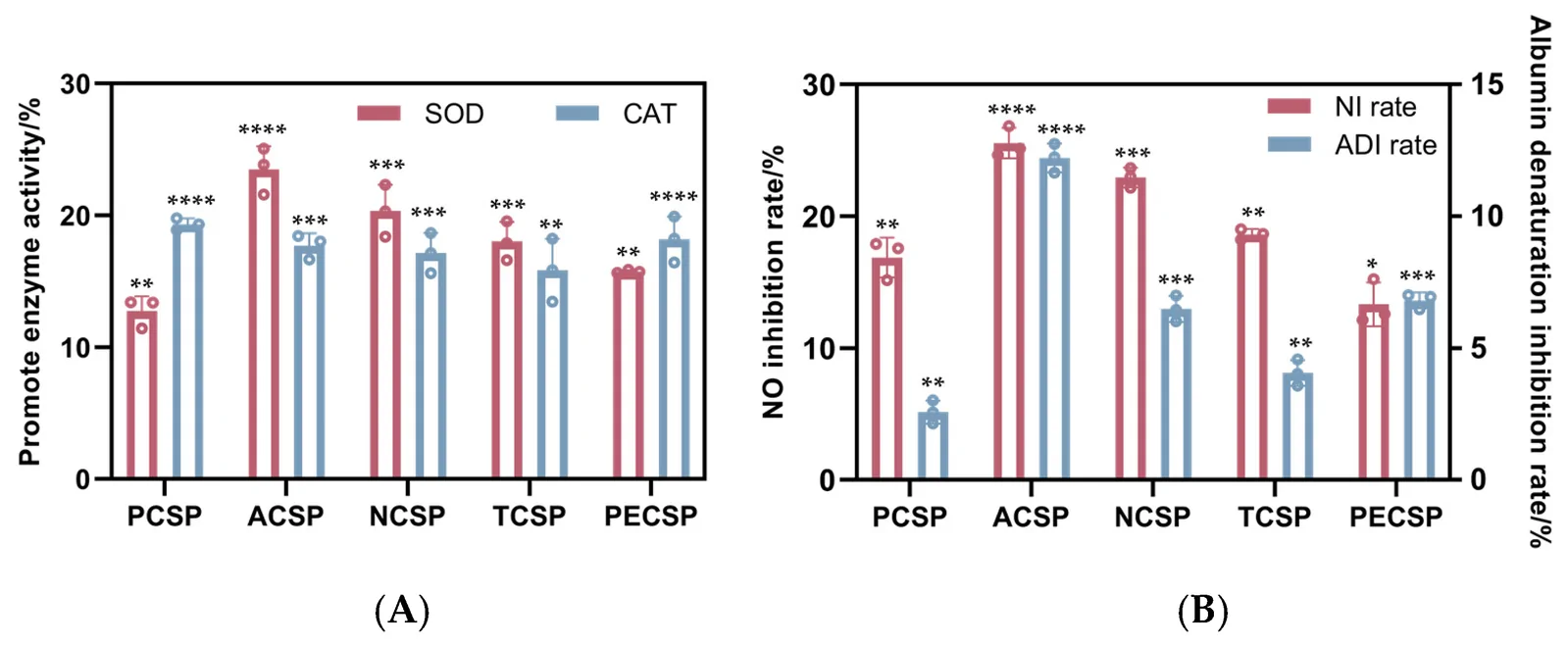
Antioxidant and anti-inflammatory activities of five CS protein hydrolysates in vitro. (**A**) Effects on SOD and CAT activities. (**B**) Inhibition of SNP-derived NO release in a cell-free system and heat-induced albumin denaturation. Data are presented as the mean ± SD (*n* = 3). Each assay was analyzed separately using one-way ANOVA followed by Tukey’s multiple-comparisons test. (*) indicates a comparison of concentrations within a group, * *p* < 0.05, ** *p* < 0.01, *** *p* < 0.001, **** *p* < 0.0001.

**Figure 2:**
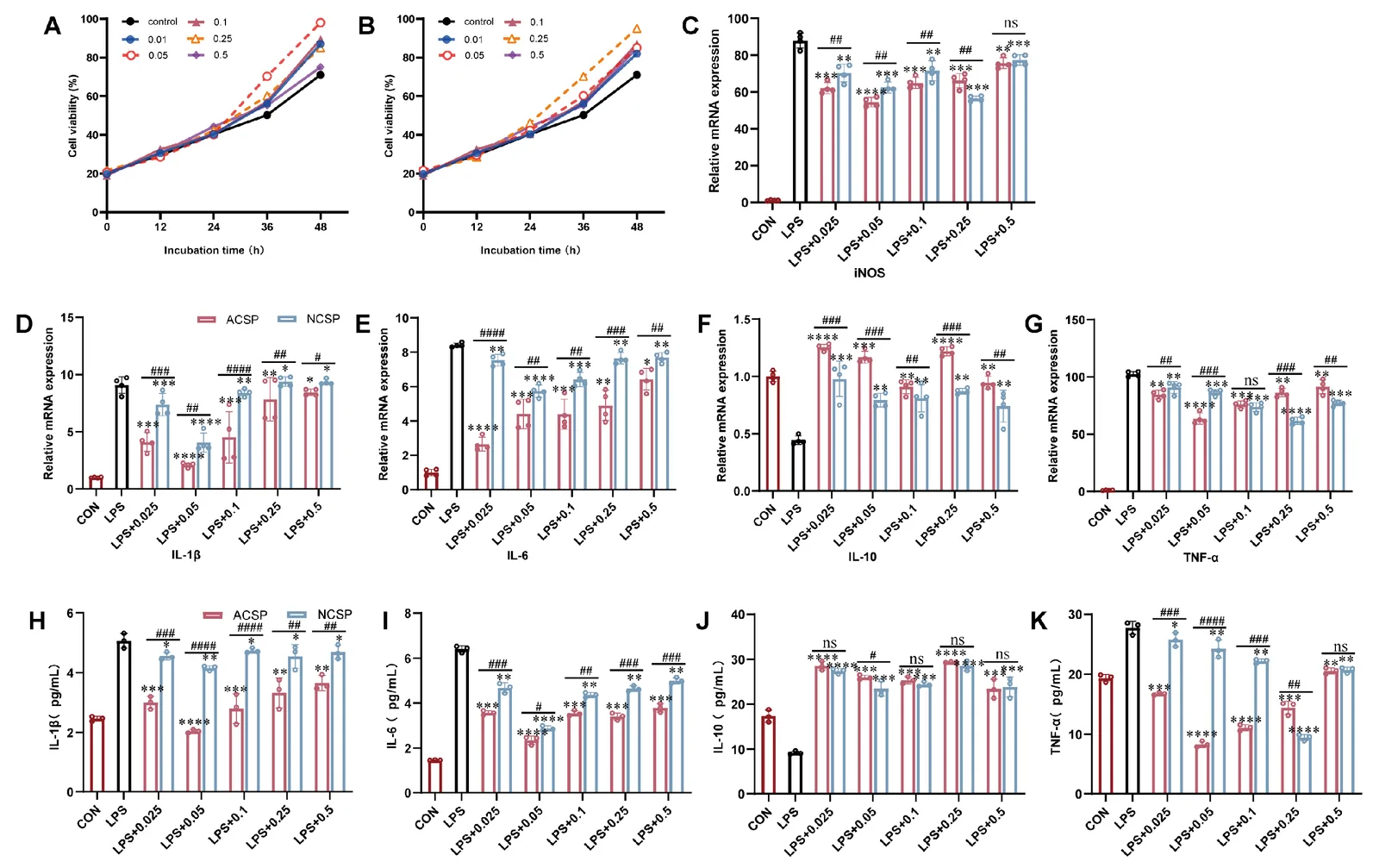
Effects of ACSP and NCSP on IEC-6 cell viability and LPS-induced inflammatory responses. (**A**,**B**) Time courses of cell viability after treatment with ACSP and NCSP, respectively, at the indicated concentrations. (**C**–**G**) Relative mRNA expression of iNOS, IL-1β, IL-6, IL-10, and TNF-α after LPS stimulation for 4 h followed by ACSP or NCSP treatment at 0.025, 0.05, 0.10, 0.25, or 0.50 mg/mL for 24 h. (**H**–**K**) IL-1β, IL-6, IL-10, and TNF-α concentrations in culture supernatants. Data are presented as the mean ± SD. Panels A and B: *n* = 3 independent experiments, with three technical wells per condition in each experiment; panels (**C**–**G**): *n* = 4 independent experiments; panels (**H**–**K**): *n* = 3 independent experiments. In panels (**C**–**K**), red and blue denote ACSP and NCSP, respectively. The viability time courses were analyzed using two-way analysis of variance. Panels (**C**–**K**) were analyzed using one-way ANOVA followed by Tukey’s multiple-comparisons test. (*) indicates a comparison of concentrations within a group; (#) indicates a comparison between groups. * *p* < 0.05, ** *p* < 0.01, *** *p* < 0.001, **** *p* < 0.0001; ns: not significant (*p* > 0.05), # *p* < 0.05, ## *p* < 0.01, ### *p* < 0.001, #### *p* < 0.0001.

**Figure 4:**
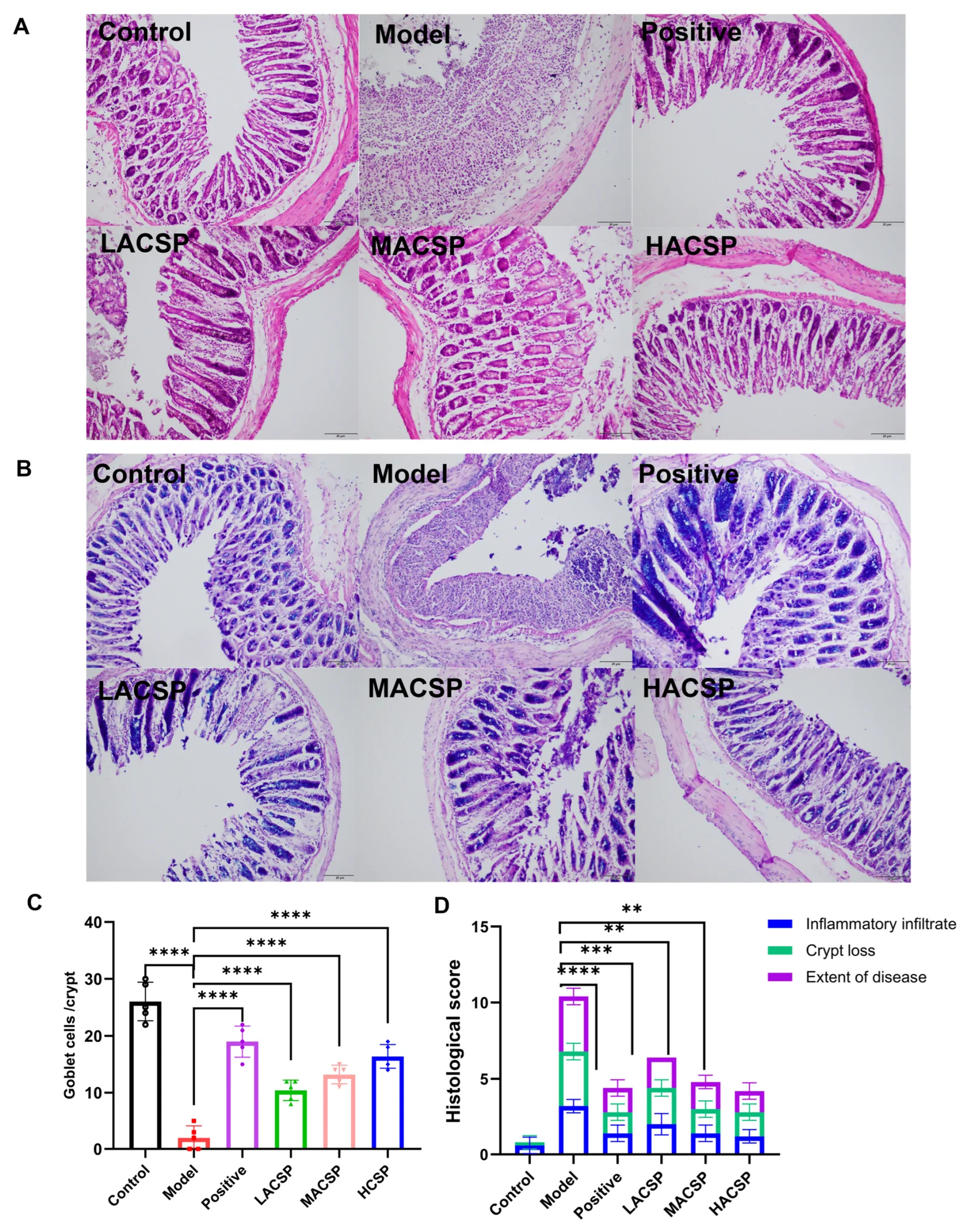
Effects of ACSP on colonic histopathology in mice with DSS-induced colitis. (**A**) Representative H&E-stained sections. (**B**) Representative AB-PAS-stained sections. (**C**) Goblet cell counts per crypt. (**D**) Histological scores. Data are presented as the mean ± SD (*n* = 5). (**C**,**D**) were analyzed separately using one-way ANOVA followed by Tukey’s multiple-comparisons test. ** *p* < 0.01, *** *p* < 0.001, **** *p* < 0.0001.

**Figure 5:**
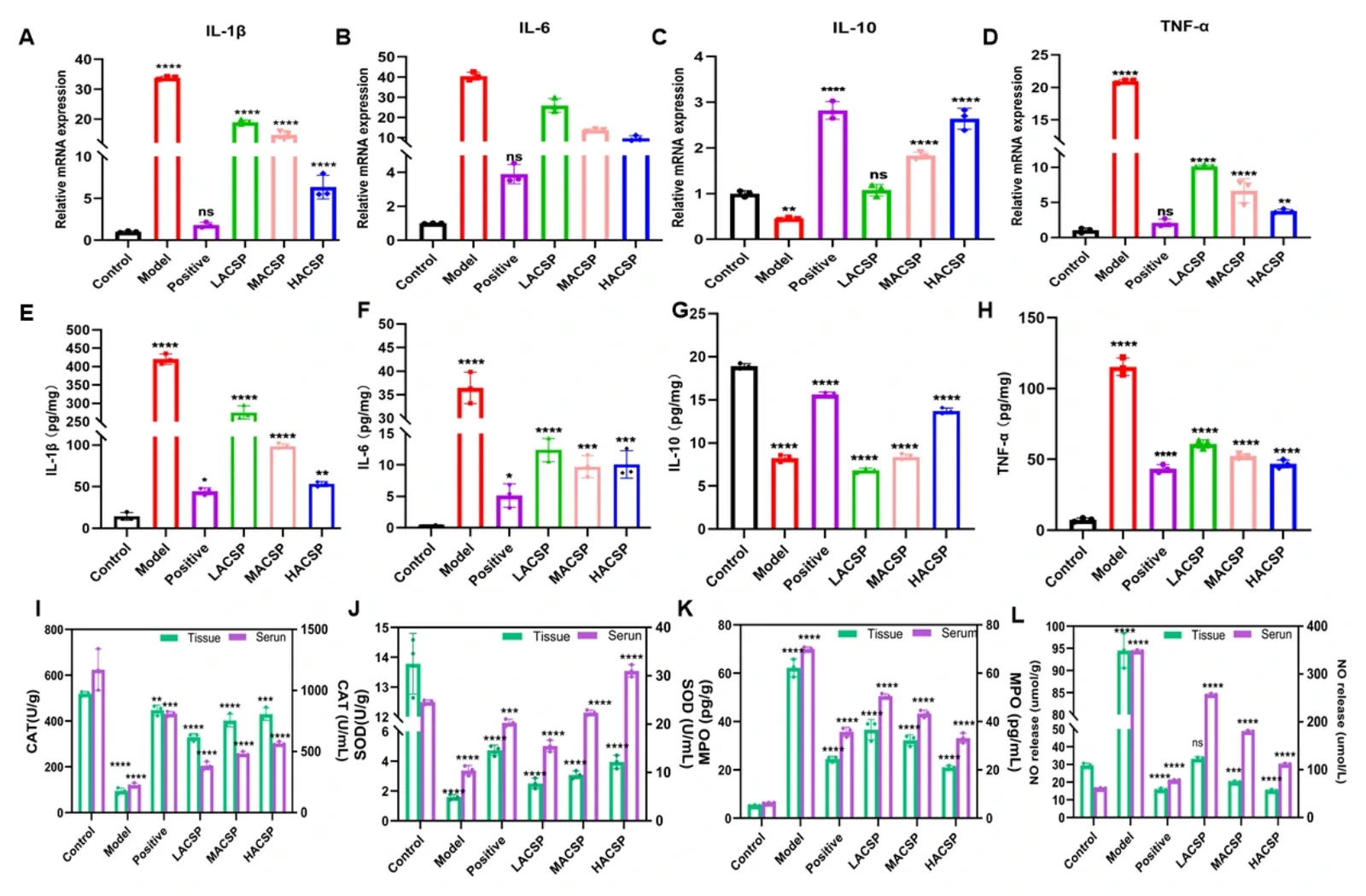
Effects of ACSP on inflammatory and oxidative stress markers in mice with DSS-induced colitis. (**A**–**D**) Relative mRNA expression of IL-1β, IL-6, IL-10, and TNF-α, respectively. (**E**–**H**) Colonic concentrations of IL-1β, IL-6, IL-10, and TNF-α, respectively. (**I**–**K**) CAT, SOD, and MPO activities in colon tissue and serum, respectively. (**L**) NO levels in colon tissue and serum. Data are presented as the mean ± SD (*n* = 3 mice per group, randomly selected from the five animals before the assays). Each biological sample was measured in technical triplicate, and technical replicates were averaged before statistical analysis. Each quantitative endpoint was analyzed using one-way ANOVA followed by Tukey’s multiple-comparisons test. * *p* < 0.05, ** *p* < 0.01, *** *p* < 0.001, **** *p* < 0.0001.

**Figure 6:**
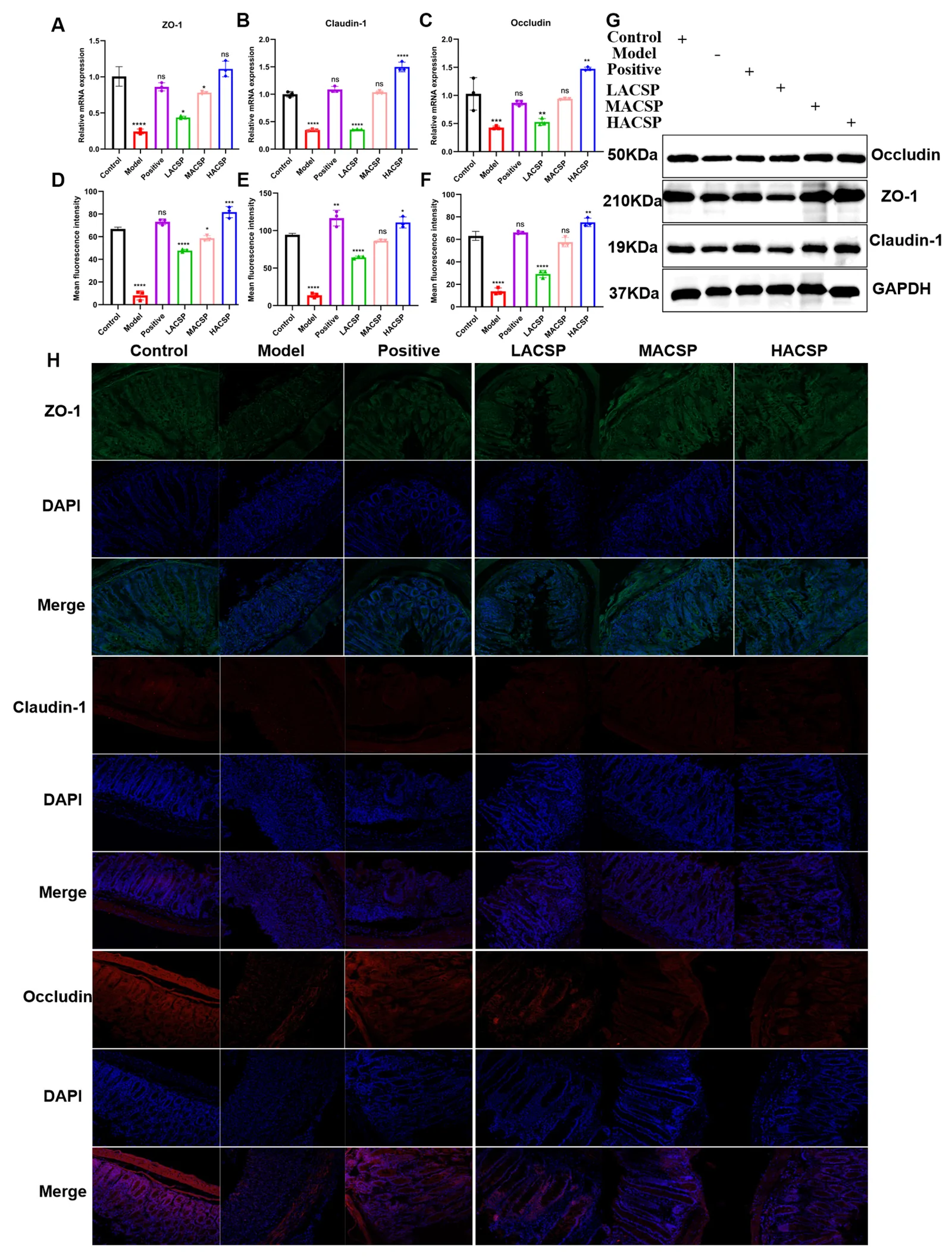
Effects of ACSP on colonic tight junction markers in mice with DSS-induced colitis. (**A**–**C**) Relative mRNA expression of ZO-1, claudin-1, and occludin, respectively. (**D**–**F**) Mean fluorescence intensities of ZO-1, claudin-1, and occludin, respectively. (**G**) Representative Western blots of ZO-1, claudin-1, and occludin in colon tissue. (**H**) Representative immunofluorescence images. Data are presented as the mean ± SD (*n* = 3 mice per group, randomly selected from the five animals before the assays). Each biological sample was measured in technical triplicate, and technical replicates were averaged before statistical analysis. Quantitative outcomes in panels (**A**–**F**) were analyzed using one-way ANOVA followed by Tukey’s multiple-comparisons test. * *p* < 0.05, ** *p* < 0.01, *** *p* < 0.001, **** *p* < 0.0001.

**Figure 7:**
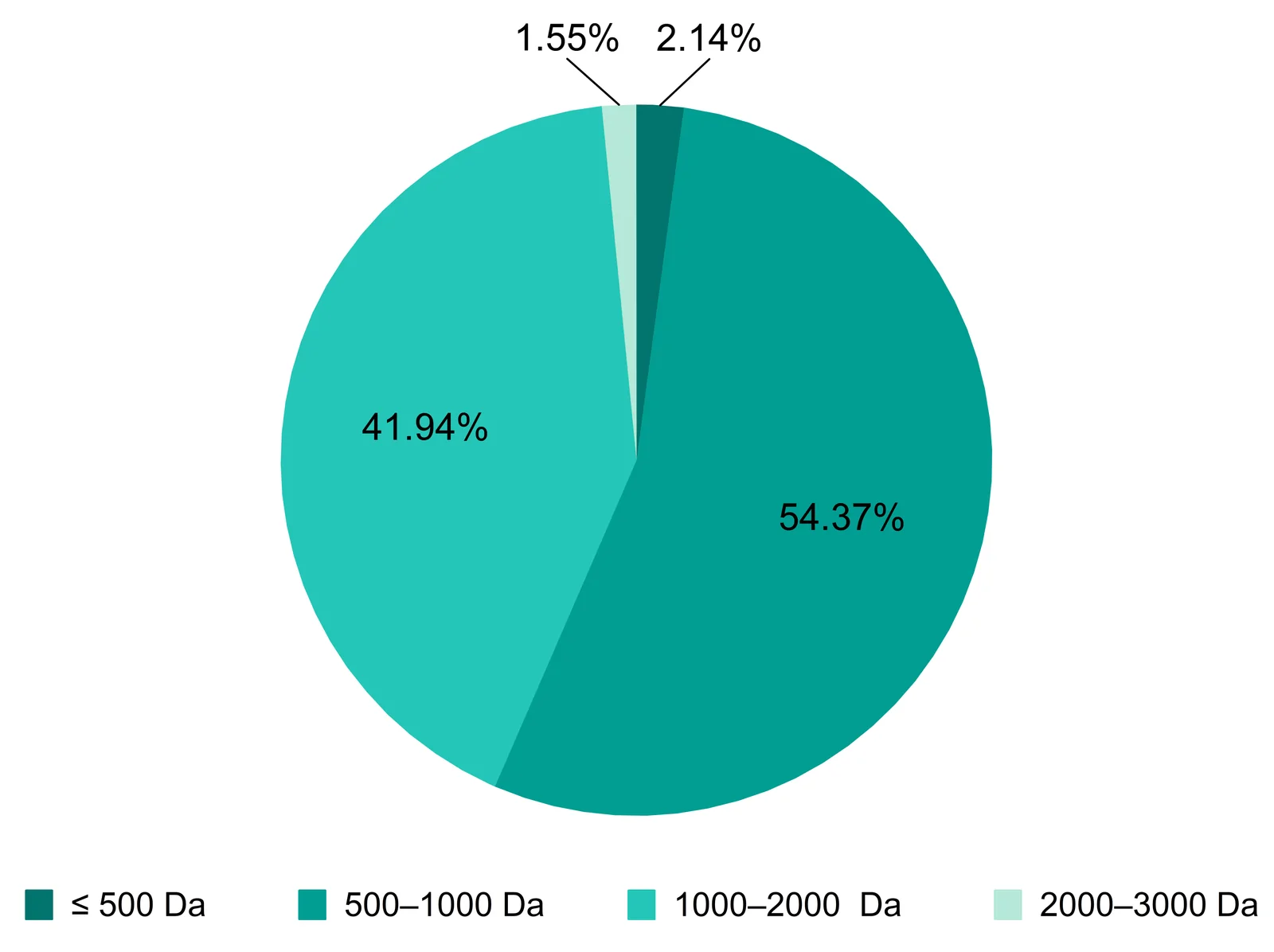
Molecular mass distribution of the 516 peptide sequences identified in ACSP by LC–MS/MS, expressed as percentages of the total sequence count.

**Table 1:** Enzymatic hydrolysis conditions used to screen the five proteases.

Protease	pH	Temp. (°C)	Time (h)	Enzyme Dose (mg/100 mL)	Substrate Concentration (g/100 mL)	Enzyme Activity Loading (U/g Substrate)	Enzyme-to-Substrate Mass Ratio (%)
Neutral protease (20,000 U/mg)	7.0	50	6	0.50	4	2500	0.013
Alkaline protease (200 U/mg)	8.0	55	6	4.00	4	2500	0.100
Papain (6000 U/mg)	6.5	65	6	1.67	4	2500	0.042
Pepsin (2500 U/mg)	3.0	40	6	4.00	4	2500	0.100
Trypsin (250 U/mg)	8.0	37	6	40.00	4	2500	1.000

**Table 2:** In vitro antioxidant activities of five CS protein hydrolysates.

Assay	Endpoint	PCSP	ACSP	NCSP	TCSP	PECSP
DPPH	IC_50_ (mg/mL)	2.205 ± 0.053 ^b^	0.888 ± 0.004 ^a^	1.094 ± 0.041 ^a^	3.256 ± 0.235 ^c^	3.791 ± 0.129 ^c^
ABTS	IC_50_ (mg/mL)	2.417 ± 0.018 ^b^	0.853 ± 0.003 ^a^	0.794 ± 0.081 ^a^	2.998 ± 0.110 ^b^	3.95 ± 0.018 ^c^
FRAP	μmol Fe(II) equivalents/g	26.156 ± 3.095 ^b^	28.683 ± 3.574 ^b^	47.637 ± 4.728 ^a^	21.102 ± 3.574 ^c^	8.466 ± 1.787 ^d^
•OH	Scavenging activity at 1.0 mg/mL (%)	27.310 ± 1.051 ^b^	29.525 ± 0.499 ^a^	22.107 ± 0.583 ^c^	19.188 ± 0.904 ^d^	19.436 ± 0.807 ^d^
O_2_•^−^	Scavenging activity at 1.0 mg/mL (%)	25.055 ± 0.715 ^c^	47.310 ± 1.351 ^a^	38.225 ± 1.025 ^b^	17.376 ± 0.461 ^d^	15.269 ± 0.851 ^d^

Note: Values are presented as the mean ± SD (*n* = 3). IC_50_ was defined as the hydrolysate concentration required to achieve 50% radical-scavenging activity. DPPH, 2,2-diphenyl-1-picrylhydrazyl; ABTS, 2,2′-azino-bis (3-ethylbenzothiazoline-6-sulfonic acid); FRAP, ferric reducing antioxidant power; •OH, hydroxyl radical; O_2_•^−^, superoxide anion radical; IC_50_, concentration producing 50% radical scavenging; SD, standard deviation. Lower DPPH and ABTS IC_50_ values indicate greater scavenging activity, whereas higher FRAP values and scavenging percentages indicate greater antioxidant activity. Data for each endpoint were analyzed using one-way analysis of variance (ANOVA) followed by Tukey’s multiple-comparisons test. Different lowercase superscript letters within the same row indicate significant differences among hydrolysates (*p* < 0.05); values sharing a letter are not significantly different.

**Table 3:** The ten highest-confidence peptide sequence assignments in ACSP.

No	Peptide Sequence	−10lgP	Length	Mass (Da)	Source of Peptide
1	GESDDDDVVAK	92.46	11	1148.4833	Myosin light chain 2
2	SDDDDVVAK	81.48	9	962.4193	Myosin light chain 2
3	SDLGHGIVLPPL	77.09	12	1216.6815	Hemocyanin subunit 2
4	YDESGPGIVH	75.97	10	1072.4825	Actin (Fragment)
5	LPVAPEFPHPIPQFVL	75.43	16	1799.9973	Calcification associated soluble matrix protein 2
6	LPVAPEFPHPIPDFVL	70.59	16	1786.9657	Strongly chitin-binding protein-1
7	WWKDSYGYH	70.40	9	1240.5302	Hemocyanin subunit 2
8	LPVAPEFPHPIPQF	70.20	14	1587.8448	Calcification associated soluble matrix protein 2
9	VAPEFPHPIPQ	70.17	11	1230.6396	Calcification associated soluble matrix protein 2
10	DLLPVAPEFPHPIPQ	69.99	15	1668.8875	Calcification associated soluble matrix protein 2

Note: Sequences are listed in descending order of the PEAKS −10lgP score. Higher scores indicate greater confidence in database-based sequence assignments and do not indicate peptide content or relative abundance.

## Data Availability

The original contributions presented in this study are included in the article/Supplementary Materials. Further inquiries can be directed to the corresponding author.

## Supplementary Materials

The following supporting information can be downloaded at https://www.mdpi.com/article/10.3390/foods15193389/s1: Figure S1: Total ion chromatogram of ACSP; Figure S2: LC–MS/MS identification quality-control plots; Figure S3: Representative annotated MS/MS spectra; Table S1: DAI scoring criteria; Table S2: Colonic histological injury scoring criteria; Table S3: Primer sequences; Table S4: Elution gradient; Table S5: LC–MS/MS search parameters; Table S6: Peptide identification results; Supplementary Methods: Assumptions used in the a priori sample-size calculation.

## Abbreviations

The following abbreviations are used in this manuscript:

IBD	Inflammatory bowel disease
ROS	Reactive oxygen species
RNS	Reactive nitrogen species
IL-1β	Interleukin-1β
IL-6	Interleukin-6
IL-10	Interleukin-10
TNF-α	Tumor necrosis factor-alpha
SOD	Superoxide dismutase
CS	Crayfish shell
IEC-6	Cell intestinal epithelial cell
LPS	Lipopolysaccharide
DSS	Dextran sulfate sodium
CAT	Catalase
MPO	Myeloperoxidase
NO	Nitric oxide
CCK-8	Cell counting kit-8
PCSP	Papain-derived crayfish shell protein hydrolysate
ACSP	Alkaline-protease-derived crayfish shell protein hydrolysate
NCSP	Neutral-protease-derived crayfish shell protein hydrolysate
TCSP	Trypsin-derived crayfish shell protein hydrolysate
PECSP	Pepsin-derived crayfish shell protein hydrolysate
DAI	Disease activity index
H&E	Hematoxylin and eosin staining
AB-PAS	Alcian Blue staining and periodic acid Schiff staining
iNOS	Inducible NO synthase gene
TJ	Tight junction proteins
