# Supplementary material for: Anti-Inflammatory Protein Hydrolysate Screened from Crayfish Shells Attenuates Colitis by Oxidative Stress Suppression, Inflammation Reduction and Barrier Reconstruction
Source: Foods. 2026 Sep 23;15(19):3389. doi: 10.3390/foods15193389 (PMC13664394; doi:10.3390/foods15193389)
Supplement: Supplementary file 1 [file foods-15-03389-s001.zip › foods-4533546-supplementary.pdf]

Anti-Inflammatory Protein Hydrolysate Screened from Crayfish Shells Attenuates Colitis by Oxidative Stress Suppression, Inflammation Reduction and Barrier Reconstruction

Lei Dong, Hao Wang, Yongmin Wu, Jinyang Peng, Shaohao Lai, Zexin Zhao,

Yongqi Yin, Jun Cai*

Supplementary Methods

(S1) Hydrolysate yields and peptide recoveries

After freeze-drying, each hydrolysate was weighed to determine the hydrolysate yield. Hydrolysate yield was calculated as follows:

Hydrolysate yield (%) = mass of the freeze-dried hydrolysate / mass of the decalcified crayfish shell powder subjected to hydrolysis × 100.

The total peptide content of each hydrolysate was determined using the BSA. Peptide recovery was calculated as follows:

Peptide recovery (%) = total peptide mass recovered in the soluble hydrolysate / initial protein mass in the decalcified substrate × 100.

(S2) Animals and experimental design

(S2.1) Sample Size Calculation Method

The sample size was determined as follows: An a priori power analysis for a one-way ANOVA with six independent groups was performed using G*Power 3.1. Assuming a large effect size of Cohen's f = 0.75, α = 0.05, and 80% power,

the minimum required total sample size was 29 mice. This was rounded up to 30 mice to allow for equal allocation, resulting in five mice per group. Each group included 5 mice, all of which were used for body weight, DAI, colon length, organ index, and histopathological analysis.

(S2.2) Details on Methods for Observing Animal Behavior Indicators

General clinical condition was evaluated by direct observation of spontaneous activity, posture, grooming behavior, coat appearance, and responsiveness to cage opening or gentle handling. No separate validated behavioral scale was applied; these observations were used only for routine animal-welfare monitoring and were not included in the disease activity index or statistical analyses. Food consumption was assessed by weighing the food supplied and the food remaining after 24 h, and daily food intake was calculated as the difference between these two measurements. Freshly voided feces and the perianal region were also visually inspected during the daily assessment.

(S2.3) Statistical analysis

IEC-6 cell-viability time-course data were analyzed using two-way analysis of variance, with treatment concentration and time as fixed factors, followed by Dunnett's multiple-comparisons test against the corresponding untreated control.

When the assumption of homogeneity of variance was violated but the data remained approximately normally distributed, Welch's analysis of

variance followed by the Games–Howell multiple-comparisons test was used. When the normality assumption was not satisfied, the Kruskal–Wallis test followed by Dunn's multiple-comparisons test was applied.

Table S1 Calculation Standard for Disease Activity Index

Weight loss rate (%)	Fecal characteristics	The situation of fecal blood	Score
0	Normal	Normal	0
1-5			1
6-10	Semiformed stool	Fecal occult blood	2
11-15			3
＞15	Loose stools	Visible blood in the stool	4

Table S2 Calculation Standard for degree of colonic injury

Score	Assessment item		
	Inflammatory infiltration depth	Crypt damage	Lesion area extent (%)
0	No infiltration	No damage	0
1	Mucous layer	Basal 1/3 recess destruction	1-25
2	submucosa	Basal 2/3 recess destruction	26-50
3	Muscular layer and serous layer	Only the complete surface epithelium	51-75
4	——	Total destruction of crypts and epithelium	76-100

Note:The total DAI was calculated for each mouse as the sum of the body-weight-loss, stool-consistency, and rectal-bleeding scores. Each component ranged from 0 to 4, resulting in a total DAI range of 0–12.

Table S3 Primer sequences used for quantitative real-time PCR.

mouse	Forward	Reverse

	GAPDH	5′-GTGACGTTGACATCCGTAAAGA-3′	5′-GTAACAGTCCGCCTAGAAGCAC-3′
	IL-1β	5′- GACTGTTTCTAATGCCTTCCC-3′	5′- CATGGTTTCTTGTGACCCTGA-3′
	IL-6	5′- CTCCCAACACGACCTGTCTATAC-3′	5′-CCATTGCACAACTCTTTTCTCA-3′
	IL-10	5′-TCCCTGGGTGAGAAGCTGAAGA-3′	5′-CACCTGCTCCACTGCCTTGC-3′
	TNF α	5′- GCGACGTGGAACTGGCAGAAG-3′	5′-GCCACAAGCACGGAATGAGAAGA-3′
	ZO-1	5'-CAGCCTGGAACAGGAGAAGA-3'	5'-GTCTGGCGATCTCGTAGTCC-3'
	Claudin	5'-TGGCTGTGATGAGCAGTTCT-3'	5'-TGGTGATGGTGATGAGGA-3'
	Occludin	5'-TCCTCCTCACCTTCACCATC-3'	5'-TGGCAACCTGCTCCTTACTT-3'
	iNOS	5′-ATCTTGGAGCGAGTTGTGGATTG-3′	5′-TAGGTGAGGGCTTGGCTGAGTG-3′
Rat	GAPDH	5′-AGGCACCAAGATACTTACAAAA-3′	5′-GGTGGTGAACTTGTTTTGCGA-3′
	IL-1β	5′-ATCCTCTCCAGTCAGGCTTCCTTG	5′-AGCTCTTGTCGAGATGCTGCTGTG-3′
	IL-6	5′-ACTTCCAGCCAGTTGCCTTCTTG-3′	5′-TGGTCTGTTGTGGGTGGTATCCTC-3′
	IL-10	5′-CCAGTCAGCCAGACCCACATGC-3′	5′-GGTTCTCTGCCTGGGGCATCAC-3′
	TNF α	5′-ATGGGCTCCCTCTCATCAGTTCC-3′	5′-CCTCCGCTTGGTGGTTTGCTAC-3′
	iNOS	5′-AAATCCTACCAAGGTGACCTGA-3′	5′-CCTGTGTTGTTGGGCTGGGAATAG-3′

Table S4 Mobile phase parameters

**Time(min)**	**Row(nl/min)**	**A%**	**B%**
0.00	250	95	5
8.00	250	90	10
33.00	250	85	15
43.00	250	72	28
50.00	250	60	40
60.00	250	5	95
65.00	250	5	95

66.00	250	95	5
70.00	250	95	5

Table S5 PEAKS database-search parameters and identification-filtering criteria used for ACSP peptidomic analysis

Category	Parameter	Setting
**Software and database**	Search software	PEAKS Studio 11.5, build 20230821 (Bioinformatics Solutions Inc.)
	Search workflow	PEAKS DB database search
	Protein sequence database	UniProtKB *Procambarus clarkii* protein database
	Taxonomy identifier	Taxonomy ID 6728
	Database file	uniprotkb_taxonomy_id_6728_ 2025_06_16. fasta
	Number of searched entries	916
	FDR estimation strategy	Target-decoy strategy; FDR estimation enabled
**Search settings**	Precursor mass type	Monoisotopic
	Enzyme specificity	None
	Digestion mode	Unspecific
	Maximum missed cleavages	3
	Peptide length range	3-45 amino acids
	Precursor-ion mass tolerance	10 ppm
	Fragment-ion mass tolerance	0.02 Da
	Fixed modifications	None specified
	Variable modifications	Protein N-terminal acetylation (+42.01 Da); oxidation of histidine/tryptophan (H/W, +15.99 Da)
	Maximum variable PTMs per peptide	2
	Deep-learning boost	No
**Identification filtering**	Peptide confidence threshold	PEAKS peptide -10lgP >= 37.04
	Protein confidence threshold	PEAKS protein -10lgP >= 13.01
	Minimum unique peptides per protein	>= 1
	De novo score threshold	>= 50%
	PSM-level FDR	1.0%
	Peptide-sequence-level FDR	1.4%
	Protein-group-level FDR	1.8%

Category	Parameter	Setting
**Quality control and reporting**	Quality-control metrics	Target-decoy FDR curve; peptide -10lgP score distribution; score-versus-precursor-mass-error plot; precursor-mass-error distribution; and precursor m/z-versus-mass-error plot (Figure S2)
	Representative spectral evidence	Annotated MS/MS spectra with matched b- and y-type fragment ions for selected high-confidence peptide sequence assignments (Figure S3)
	Complete identification output	Complete PEAKS search report and peptide-identification list (Supplementary Data S1 and Table S6)

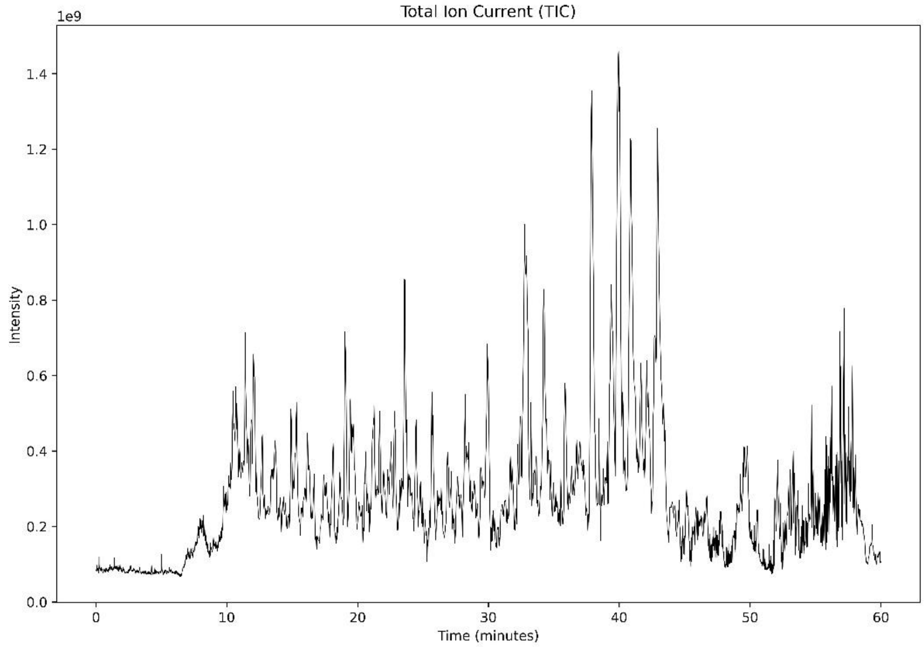

Figure S1. Total ion current plot of ACSP

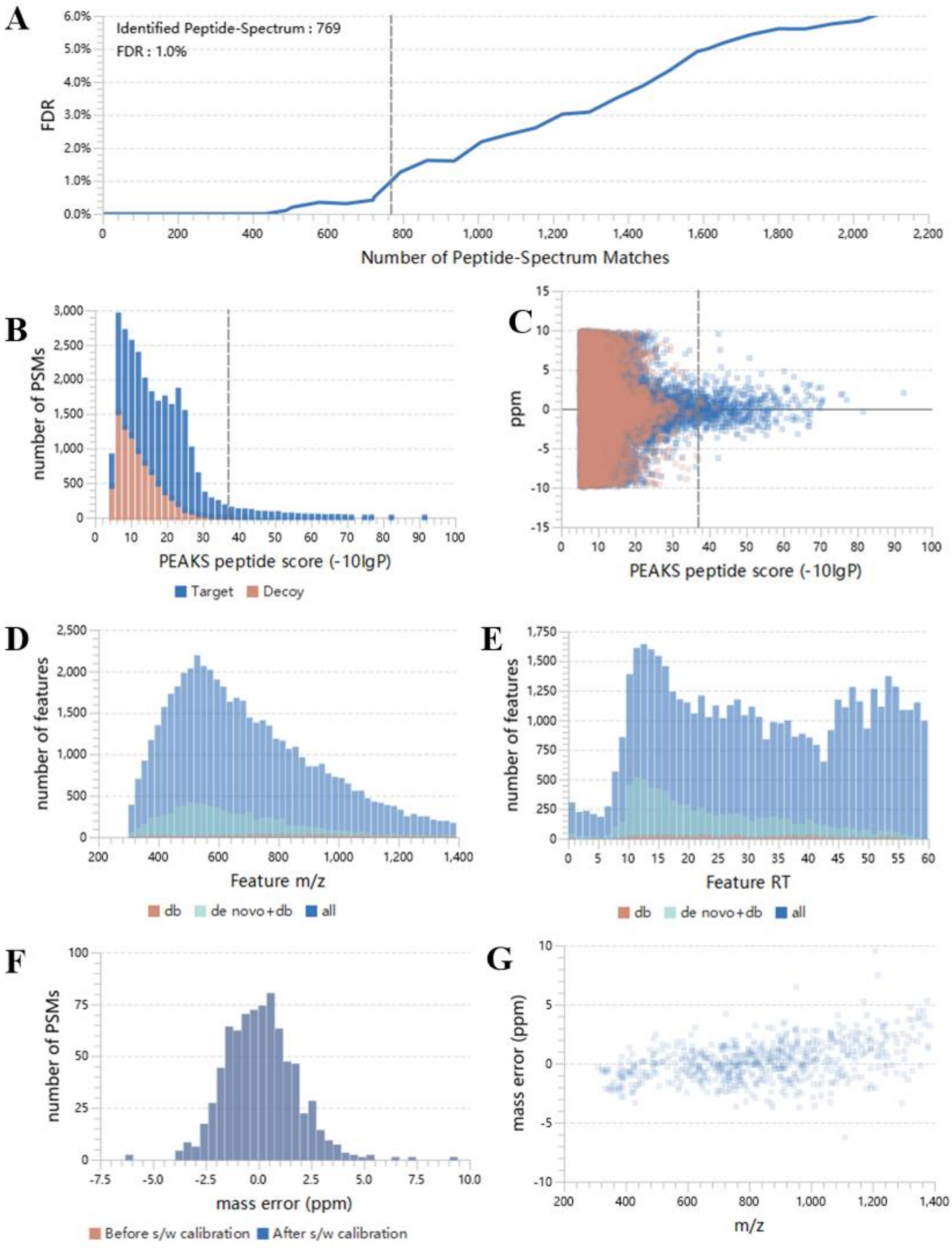

Figure S2. LC–MS/MS identification quality-control plots. (A) False discovery rate (FDR) curve. X axis is the number of peptides being kept. Y axis is the corresponding FDR. (B) Distribution of PEAKS peptide score. (C) Scatterplot of PEAKS peptide score versus precursor mass error. (D) Feature m/z distribution. (E) Feature RT distribution. (F) Distribution of precursor mass error in ppm. (G) Scatterplot of precursor m/z versus precursor mass error in ppm.

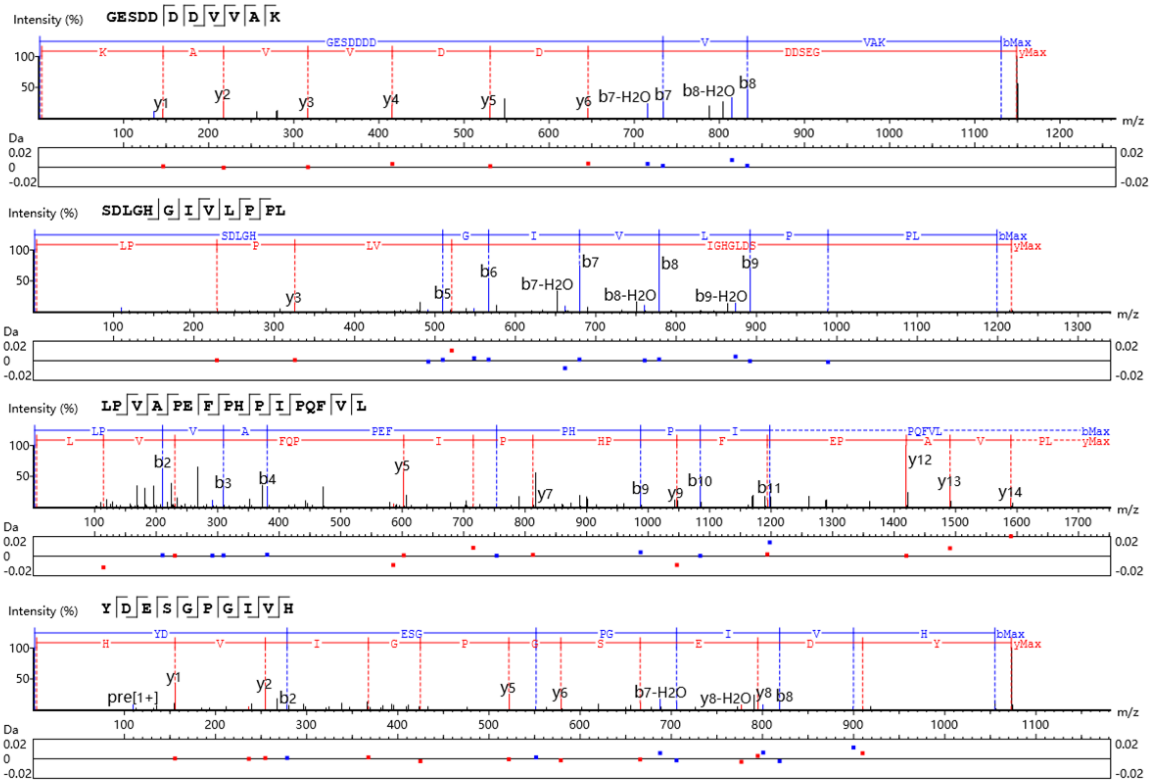

Figure S3. Representative annotated MS/MS spectra.

Table S6 Peptide identification results

Peptide	-10LgP	Mass	Length	ppm	m/z	RT	Scan
GESDDDDVVAK	92.46	1148.4833	11	2.1	1149.49292	11.3209	3905
SDDDDVVAK	81.48	962.4193	9	-0.4	963.42615	10.559	3505
SDLGHGIVLPPL	77.09	1216.6815	12	1.1	1217.69019	38.4595	17168
YDESGPGIVH	75.97	1072.4825	10	1.9	1073.49182	15.677	6026
LPVAPEFPHPIPQFVL	75.43	1799.9973	16	1.8	901.00757	52.6831	23466
LPVAPEFPHPIPDFVL	70.59	1786.9657	16	1.2	894.49121	52.1568	23220
WWKDSYGYH	70.4	1240.5302	9	0.9	1241.53857	20.9995	8608
LPVAPEFPHPIPQF	70.2	1587.8448	14	0.8	794.93036	41.2383	18521
VAPEFPHPIPQ	70.17	1230.6396	11	2.7	1231.65027	26.3691	11225
DLLPVAPEFPHPIPQ	69.99	1668.8875	15	0	835.45105	41.7091	18789
LLPVAPEFPHPIPQ	69.53	1553.8606	14	0.2	777.93774	36.7044	16194

Peptide	-10LgP	Mass	Length	ppm	m/z	RT	Scan
APEFPHPIPQ	69.32	1131.5713	10	2.7	1132.58154	24.6511	10445
GHGIVLPPLYE	68.59	1193.6444	11	1.2	1194.65308	33.6285	14730
EQHHWFSLF	67.58	1229.5618	9	1.6	1230.57104	38.5467	17170
YWDHPIRDGFAPH	67.33	1609.7426	13	-0.1	805.87848	22.5071	9344
AELEHILLSLGERLE	67.28	1720.9359	15	3	861.47784	52.7851	23457
SGPGIVHR	67.21	821.4507	8	-0.7	411.73236	10.3916	3441
GHGIVLPPLY	67.13	1064.6018	10	-1.7	1065.6073	34.1482	15003
AAPAPSPT	66.94	710.3599	8	-2.3	711.36554	11.1074	3779
DDDDVVAK	66.86	875.3872	8	3	876.39709	10.559	3535
DLGHGIVLPPL	66.74	1129.6495	11	1.7	1130.65869	41.3663	18533
FPHPIPQFVL	66.55	1193.6597	10	-2.1	1194.66443	40.8952	18376
KYLSKSIFD	66.55	1099.5913	9	-1.1	1100.59741	20.5687	8406
TEAPLNPK	66.44	868.4654	8	-0.6	869.47217	11.873	4216
KSYELPDGQVI	66.18	1247.6398	11	2.3	1248.6499	28.5311	12315
YDGVPVPGSPF	65.99	1133.5393	11	1	1134.54773	37.4388	16616
SDLLPVAPEFPHPIPQ	65.72	1755.9196	16	-0.2	878.96686	39.9933	17976
VIPIPFPINPE	65.38	1234.6962	11	3.1	1235.70728	47.8829	21564
LPVAPEFPHPIPD	64.97	1427.7449	13	-1.4	714.87872	34.2767	15059
LPVAPEFPHPIPQ	64.5	1440.7765	13	0.5	721.39587	32.8526	14427
LPVAPEFPHPIPDF	63.89	1574.8132	14	-3.2	788.41138	42.9319	19242
SDLLPVAPEFPHPIPQFVL	63.88	2115.1404	19	0.3	1058.57776	53.9899	24032
SDLLPVAPEFPHPIPD	63.54	1742.8879	16	1.1	872.45215	41.6655	18728
GHGIVLPPL	63.52	901.5385	9	-1.1	902.5448	31.7218	13920
EAPLNPK	63.25	767.4177	7	-1.5	768.42389	10.6856	3539

Peptide	-10LgP	Mass	Length	ppm	m/z	RT	Scan
FPHPIPQFVLD	63.02	1308.6866	11	1.6	1309.69604	37.3114	16533
FPHPIPDFVL	62.81	1180.6281	10	4.1	1181.64014	40.5983	18124
YLSKSIFDSLK	62.51	1299.7074	11	-0.9	1300.7135	28.4877	12301
HGIVLPPL	62.45	844.517	8	-1.4	845.52313	31.6786	13921
YLSKSIFD	62.4	971.4964	8	1.1	972.50476	26.1531	11122
DLLPVAPEFPHPIPD	61.72	1655.8558	15	0.1	828.93524	43.4963	19615
DFPFWWDGAK	61.26	1267.5662	10	2.4	1268.57654	52.4066	23284
GGEFPARPDDVHFE	61.26	1571.7004	14	0.7	786.85803	24.7238	10487
HVTWHMDFPF	61.11	1315.5808	10	2.3	1316.59106	38.5467	17158
SDLLPVAPEFPHPIPDF	60.9	1889.9563	17	2.8	945.98804	50.9961	22770
IHIPGSPF	60.84	866.465	8	-1.2	867.47125	31.6302	13756
GILTLKYPIE	60.81	1145.6696	10	-1.2	1146.67554	34.2335	15226
KYLSKSIF	60.81	984.5644	8	0.2	985.5719	20.7842	8523
FDPIIEDYHK	60.6	1275.6135	10	2.1	1276.62354	23.2821	9728
TLLPWSTKF	60.42	1091.6016	9	2.7	1092.61169	36.4819	16192
SDLLPVAPEFPH	60.2	1320.6714	12	4.8	1321.68494	36.8379	16312
GIITNWDDM	59.64	1063.4645	9	-2.5	1064.46899	39.9479	17831
AKDPYGPPGKPGIPDIVDW	59.44	2021.0258	19	0.5	1011.52063	36.32	16026
HKYMDNIF	59.28	1066.4906	8	0.3	1067.49817	22.5955	9415
SDLGHGIVLPPLY	59.07	1379.7449	13	1.3	1380.75391	40.2499	17940
DFPFWWKD	59.02	1139.5076	8	-0.5	1140.51428	52.1188	23181
DHTVINPE	58.45	923.4348	8	0.6	924.44269	14.3089	5353
RLSNWLDVVDEIHWE	58.35	1909.9323	15	1	955.9743	53.1379	23644
TPLVPGDYY	58.15	1023.4913	9	-1.8	1024.4967	31.6786	13830

Peptide	-10LgP	Mass	Length	ppm	m/z	RT	Scan
ALLLVDVLLHSADFE	58.08	1653.8977	15	0.2	827.9563	55.6546	24826
VFPSIVGRPR	58.01	1126.6611	10	-1.3	564.3371	19.6578	7911
PAAAAPAPA	57.76	735.3915	9	-1.5	736.39764	13.453	4994
VTPHLFTN	57.74	927.4814	8	0.4	928.48901	27.7522	11939
KLIDDHFLF	57.69	1146.6073	9	1.1	1147.61584	34.5778	15251
VAPEFPHPIPQF	57.55	1377.7081	12	1.5	1378.71741	36.1426	15946
IDDHFLFK	57.53	1033.5232	8	1.8	1034.53235	26.6611	11392
LPVAPEFPHPIPQFVLD	57.22	1915.0243	17	1.4	958.52081	46.5838	21010
EQHHWFS	57.14	969.4093	7	0	970.41656	15.7193	6061
FNGIHIPGSPF	56.79	1184.5978	11	2.3	1185.60779	39.047	17409
SELTPHVVLPPL	56.71	1300.739	12	1	1301.74768	37.5285	16687
GGEFPARPDNVHFE	56.34	1570.7164	14	0	786.36548	23.5459	9817
HNVASFIFPL	56.32	1143.6077	10	2.1	1144.61731	42.5384	19106
YDPTGPH	56.1	785.3344	7	-1.8	786.34027	10.842	3663
GGEFPARPD	55.99	944.4352	9	-0.8	945.44165	15.3284	5938
GFAGDDAPR	55.91	904.4039	9	0.2	905.41132	12.9791	4708
KRVPFSHHDRLGFL	55.89	1707.9321	14	-2.2	570.31671	19.4495	7894
AGFAGDDAPR	55.88	975.441	10	-0.5	488.72754	13.7074	5064
WDHPIRDGFAPH	55.84	1446.6792	12	-2.4	724.34515	19.0685	7764
WHMDFPF	55.83	978.4058	7	2.8	979.41577	43.7043	19717
PVAPEFPHPIPQ	55.75	1327.6924	12	-0.6	1328.69885	40.0352	17889
FPHPIPQ	55.67	834.4388	7	-1.4	835.44495	19.6673	7891
DESGPGIVH	55.54	909.4192	9	-0.2	910.42627	13.3681	4919
TRDPSFFRLH	55.45	1274.652	10	-0.1	425.89124	22.6847	9430

Peptide	-10LgP	Mass	Length	ppm	m/z	RT	Scan
YFGEDIGMNIH	55.37	1294.5652	11	-3.4	1295.56812	31.6356	13811
KRVPFSHHDRL	55.24	1390.7582	11	-1.2	464.59277	10.4757	3468
HGIDILGDIIE	55.19	1193.6292	11	1.3	1194.63794	43.1494	19430
HGIDLLGDIIE	55.19	1193.6292	11	1.3	1194.63794	43.1494	19430
DLEFPGVVVD	55.14	1088.5389	10	1.8	1089.54822	41.9715	18876
FPHPIPQF	55.04	981.5072	8	0.9	982.51532	30.9442	13521
VPNISDWSR	55.04	1072.5302	9	-1.6	537.27148	23.624	9902
DEDGFIPY	55.01	954.397	8	1.7	955.40594	36.1	15921
KDPYGPPGKPGIPDIVDW	54.77	1949.9886	18	-0.4	976.00122	36.6179	16189
IIAPPERKY	54.74	1085.6233	9	-2.3	362.88089	14.9059	5627
WWKDSYGY	54.7	1103.4712	8	1.7	1104.48035	27.9739	12127
YVAVIH	54.66	700.3908	6	-0.2	701.39795	15.932	6150
LPIDVITSPL	54.46	1066.6273	10	1.4	1067.63611	44.1677	19816
RGILTLKYPIE	54.45	1301.7706	11	-1.2	651.89185	28.2305	12231
VPPVLDSPE	54.39	951.4913	9	-2.7	952.49597	23.5814	9855
AVLSLYASGRTT	54.33	1237.6666	12	-1.4	619.83972	23.6676	9912
RDPSFFR	54.32	923.4613	7	0.1	462.73801	16.5227	6441
HHWF	54.24	625.2761	4	-0.8	626.2829	16.2299	6276
KVIPIPFPINPE	54.08	1362.7911	12	4.5	1363.80444	39.6503	17709
IDDHFLFKE	53.95	1162.5658	9	-1.2	1163.57178	32.981	14459
DFWLDVIIT	53.93	1120.5804	9	1.3	1121.58911	54.4006	24212
WDHPIRD	53.85	937.4406	7	1.1	938.44885	13.2822	4869
HVVLPPL	53.65	773.4799	7	0.7	774.48773	27.3583	11811
FKLPGISPF	53.6	1004.5695	9	0.2	1005.5769	39.6077	17746

Peptide	-10LgP	Mass	Length	ppm	m/z	RT	Scan
FAIYPAPQ	53.43	905.4647	8	1.1	906.47296	29.3542	12696
SGDDGVQDDL	53.37	1019.4043	10	2.8	1020.41443	20.394	8324
EQHHWF	53.32	882.3773	6	-0.4	883.38416	18.0806	7178
SGPGIVH	53.32	665.3497	7	-2.1	333.6814	11.4467	3925
ALLLVDVLLH	53.29	1104.6907	10	1.1	1105.6991	53.7297	23916
DEAQSKRGIL	53.23	1115.5935	10	0	558.80402	13.75	5104
TRDPAFFRLH	53.02	1258.6571	10	0.8	420.55997	23.0246	9627
YVAVTHSELTPHVVLPPL	53	1971.0829	18	1.2	986.54993	36.2294	16031
AEHDDKVTVR	52.92	1168.5836	10	-2.1	390.53433	8.1534	2438
SDLGHGIVLPPLYE	52.88	1508.7875	14	-2.3	755.39929	39.4359	17533
WRPPVYDGGLPVTH	52.69	1592.8099	14	0.5	797.41266	28.1015	12073
GEDIGMN	52.66	734.2905	7	-1.5	735.29669	14.9479	5668
DRPIPDLR	52.33	980.5403	8	-0.8	327.85379	17.1356	6764
DRKGELFFWVH	52.23	1432.7251	11	1.3	717.37079	33.8889	14912
VTPIEPA	51.95	725.3959	7	0	726.4032	17.4369	6879
SPLVIPDIIVL	51.91	1177.7322	11	1.9	1178.7417	54.7488	24371
PGAPGAPR	51.87	721.3871	8	-1.3	722.39343	8.9954	2843
DRPIPDLRVF	51.79	1226.6771	10	1.1	614.3465	30.9875	13463
LLPVAPEFPHPIPD	51.78	1540.8289	14	0	771.42175	38.1996	17042
RDPSFFRLH	51.77	1173.6042	9	-2.8	392.20761	22.853	9517
FDVPVF	51.68	722.3639	6	0.6	723.37158	41.5794	18585
GELFFWVH	51.65	1033.5021	8	-1.6	1034.50769	43.4529	19538
FIPIFAQVK	51.56	1061.6273	9	2.6	1062.63733	34.9679	15397
RVPFSHHDRL	51.47	1262.6632	10	-0.6	421.89474	11.9591	4327

Peptide	-10LgP	Mass	Length	ppm	m/z	RT	Scan
KSYELPDGQ	51.45	1035.4873	9	1.1	1036.49573	16.6948	6530
VAPEFPHPIPD	51.36	1217.608	11	0.6	1218.61609	27.8429	11910
DLLYSNVKIT	51.33	1164.639	10	1.3	1165.64783	31.204	13570
DPVLFPSFIH	51.31	1170.6073	10	0.9	1171.6156	41.1522	18504
APKFTVPL	51.25	871.5167	8	0.8	872.52472	28.9204	12506
WKDSYGY	51.19	917.3919	7	-2.7	918.39673	19.0682	7687
HTDAIPDPK	51.03	992.4927	9	2.5	497.25485	11.7494	4119
FFWVH	51.02	734.354	5	-0.3	735.36108	31.2472	13587
YIGEPQPD	50.97	917.413	8	-0.4	918.41992	17.134	6737
APEFPHPIPDF	50.93	1265.608	11	-0.2	1266.61511	36.3151	16062
IKIIAPPE	50.92	879.5429	8	0.5	880.55066	22.0367	9134
KYMDNIF	50.88	929.4317	7	-1.7	930.43738	28.3164	12193
IRDPVLFPSFIH	50.84	1439.7925	12	1.5	720.9046	35.6276	15717
SDLLPVAPEFPHPIPQF	50.84	1902.9879	17	6.5	952.50739	48.9326	21880
WFDWK	50.83	780.3595	5	0.8	781.36737	30.8456	13417
KIIAPPE	50.77	766.4589	7	-1	767.46539	15.207	5796
EPPAPKP	50.67	734.3962	7	-0.8	735.40295	8.9822	2696
GHPPVFTIPF	50.61	1110.5862	10	-0.9	1111.59241	39.7794	17788
LWPPPPPPVVSPT	50.56	1382.7598	13	3.2	1383.77148	36.0562	15982
VAPEFPHPIPDF	50.55	1364.6764	12	3.9	1365.68909	37.4417	16607
VNVDPDGK	50.52	842.4134	8	-1	843.4198	11.3635	3935
KTFFDTF	50.49	904.433	7	0.9	905.44116	34.9245	15416
DEDGFIPYEPF	50.46	1327.5608	11	2	1328.57068	51.886	23072
FPHPIPD	50.43	821.4072	7	-1.7	822.41302	21.1278	8751

Peptide	-10LgP	Mass	Length	ppm	m/z	RT	Scan
SDLLPVAPEFPHPIPDFVL	50.39	2102.1086	19	0.6	1052.06226	54.1771	24185
HTVPIYE	50.36	857.4283	7	0.4	858.43591	16.8249	6589
GGPISIPVGPGTL	50.32	1163.655	13	0.7	1164.66309	39.736	17710
GIHIPGSPF	50.3	923.4865	9	-1.4	924.49249	33.0241	14535
HHWFSLF	50.24	972.4606	7	0.1	973.46796	38.1939	16960
IDDHFLF	50.01	905.4283	7	-0.2	906.43536	36.6611	16236
HMDFTGT	49.99	807.3221	7	-2.6	808.32727	14.9479	5695
IVLPPLYE	49.84	942.5426	8	-0.8	943.54907	39.2547	17536
NVASFIFPL	49.81	1006.5488	9	2.8	1007.55884	52.2678	23223
TRDPSFFRL	49.73	1137.593	9	-1	380.20459	29.007	12530
IRDPVLFPS	49.64	1042.5811	9	0.1	1043.5885	28.3179	12191
EEFIPIF	49.6	893.4534	7	2.4	894.46283	52.6625	23421
EDNVVPEVPE	49.58	1125.5189	10	-1.3	1126.52478	26.2439	11152
GFNPPDLDIM	49.43	1117.5114	10	-1.7	1118.51672	42.1865	18910
VVLPPLYE	49.33	928.5269	8	-0.1	929.53412	36.2718	16005
GIITNW(+15.99)DDME	49.29	1208.502	10	1.8	1209.51135	34.0198	14973
SKRGILTLKYPIE	49.25	1516.8977	13	-1.4	759.45508	23.6244	9993
REELPAEKLQIFYEVLVTAGTEPAIR	49.21	2971.6018	26	1.7	991.54285	53.5518	23842
VAYWFDWKVT	49.21	1313.6444	10	-1.4	1314.6499	43.3661	19485
SDLEFPGVV	49.2	961.4756	9	-3.7	962.47931	37.8975	16815
EFPHPIPQ	48.99	963.4814	8	-1.5	964.48718	21.735	8985
GIVLPPLY	48.97	870.5215	8	0.6	871.52924	42.3455	19092
SLFQPSFL	48.87	937.4909	8	0.4	938.4986	49.7776	22248
EQHHWFSL	48.86	1082.4934	8	1.8	1083.50256	27.4971	11853

Peptide	-10LgP	Mass	Length	ppm	m/z	RT	Scan
KDSLPPY	48.82	818.4174	7	0.2	410.21606	17.2652	6804
SPGGVTE	48.77	645.2969	7	-1.7	646.3031	10.6856	3461
VVLPPLY	48.74	799.4844	7	0.8	800.49225	37.3981	16675
HGIVLPPLY	48.66	1007.5804	9	-1.8	1008.58588	34.1482	15032
FAGDDAPR	48.42	847.3824	8	-0.2	424.69839	11.702	4093
SDDDDVVA	48.4	834.3243	8	-0.4	835.33124	13.7074	5080
WWKDSYGYHL	48.39	1353.6143	10	-0.2	1354.62122	28.4019	12237
FQTNLVPYPR	48.35	1233.6506	10	-0.2	617.83246	26.1201	11142
IPFIGNPRPR	48.3	1165.672	10	-0.4	583.84302	22.853	9428
KDPWDEPGKIL	48.25	1296.6714	11	-0.7	649.34253	27.7134	11937
SDLLPVAPEFPHPIPQFVLD	48.14	2230.1672	20	2.3	1116.09351	53.007	23559
WWDGAKID	47.97	989.4606	8	0.6	990.46857	27.5836	11833
KEYEPE	47.93	793.3494	6	-0.6	794.3562	9.9682	3217
GYPVDRRIPDNRVF	47.89	1702.8903	14	-0.9	852.45166	25.7643	10908
HVTWH	47.87	678.3238	5	-0.7	340.16891	10.0942	3277
SSVYSPN	47.87	752.3341	7	0.7	753.34186	13.0278	4725
YMDNIFK	47.83	929.4317	7	-1.4	930.43768	25.6325	10883
YTPLVPG	47.8	745.401	7	2	746.40979	24.1832	10180
FFRLH	47.79	718.3915	5	-1.5	360.20245	16.6116	6507
PEFPHPIPQ	47.67	1060.5342	9	-3.3	1061.53796	37.8975	16822
GYITDK	47.55	695.349	6	-2.3	696.35468	11.4854	3982
GVNLPGVPVDLPAV	47.51	1345.7605	14	3.9	1346.77307	47.0632	21152
DLLPVAPE	47.43	852.4592	8	-1.5	853.46521	32.8526	14411
KLPGISPF	47.32	857.501	8	-3.2	858.50562	29.915	13050

Peptide	-10LgP	Mass	Length	ppm	m/z	RT	Scan
TQTPGKFH	47.27	914.461	8	0.2	915.46844	10.2007	3343
VTSPGGVTE	47.26	845.413	9	0.5	846.42072	13.75	5107
GIITNW(+15.99)DDM	47.17	1079.4594	9	-1.6	1080.46484	32.8949	14400
FWVH	47.16	587.2856	4	1.1	588.29352	24.8954	10523
HIPGSPF	47.13	753.3809	7	1.3	754.38922	24.1399	10161
SYDDIK	47.1	739.3388	6	-0.6	740.34564	11.9591	4232
DLEFPGVVVDGVAIDGELK	46.94	1971.02	19	-0.1	986.51715	45.8785	20640
GFPFDRPIPDL	46.91	1272.6503	11	-0.4	637.33215	41.1956	18833
QGPVIPR	46.83	765.4497	7	-0.9	383.73178	14.4798	5448
WDDMEKIW	46.8	1121.4851	8	0.8	1122.49329	33.9755	14945
RLSNWLDVVDE	46.76	1344.6674	11	0.2	673.34106	37.635	16698
QHHWF	46.7	753.3347	5	-0.2	754.3418	16.6091	6461
TGRWVPV	46.62	813.4497	7	-0.7	407.73184	21.65	8960
DFPNLPY	46.58	864.4017	7	0.3	865.4093	38.4502	17107
TIITYPW	46.57	892.4694	7	-0.4	893.47632	40.1219	17937
MPPWVNF	46.53	889.4156	7	0.6	890.4234	43.9731	19902
TIPEPFPITH	46.53	1150.6022	10	2.6	1151.61255	30.3314	13177
AIFGISGIYLPK	46.52	1277.7383	12	1	1278.74683	42.3022	18980
DLLPVAPEFPHPIPDF	46.52	1802.9243	16	1.9	902.47113	52.3398	23268
FLPMPT	46.52	704.3567	6	-2.3	705.36237	29.9965	13001
GPGIVH	46.44	578.3176	6	-1.5	579.32404	11.4058	3946
KDFGDVN	46.44	793.3606	7	0	794.36786	12.2568	4344
VHIKLPKLAA	46.34	1088.7069	10	-0.9	1089.71326	22.5531	9406
IIAPPERKYS	46.26	1172.6553	10	-1.1	391.89194	14.3506	5376

Peptide	-10LgP	Mass	Length	ppm	m/z	RT	Scan
RIPDNRVF	46.12	1015.5563	8	0	508.78543	17.5226	7176
TQTPGKF	46.05	777.4021	7	-1.4	389.70776	12.6824	4556
VDPDGKF	46.04	776.3704	7	-0.7	777.37714	13.6655	5045
AVFPSIVGRPR	46	1197.6982	11	-0.7	599.85596	21.735	9070
GLEPGHQY	45.85	899.4137	8	-1.1	900.41998	14.0059	5218
LLPVAPEFPHPIPQFVL	45.85	1913.0814	17	2.5	957.55035	53.7069	23890
FTPIYDF	45.84	901.4221	7	-0.5	902.42902	40.1655	17936
KYPDKRPM	45.79	1033.5378	8	-0.5	345.51971	10.2661	3378
SIISFMKGPL	45.73	1091.6049	10	-0.4	1092.61169	36.4819	16054
DKIKDPEFR	45.59	1146.6033	9	-0.5	383.20819	12.8108	4641
GGPISIPVGPG	45.59	949.5233	11	0.2	950.5307	31.0308	13483
HDFGIVIL	45.57	912.5069	8	-2.1	913.51227	37.8742	16840
GYPVDR	45.56	705.3445	6	-2.3	353.67874	11.4087	3917
FIRDPVLFPS	45.53	1189.6495	10	0.5	595.83234	33.1106	14513
FLPMPTL	45.49	817.4408	7	-1.6	818.44672	42.8093	19302
RVPTPDVS	45.47	869.4606	8	-0.5	435.7374	14.3089	5297
IDDHFL	45.4	758.3599	6	0.7	759.36768	22.51	9388
ALFQPSFL	45.37	921.496	8	2.6	922.50562	50.169	22497
SDLGHGI	45.34	697.3395	7	-0.4	698.3465	15.1627	5772
HILLSLGERLE	45.22	1278.7295	11	1.7	1279.73901	29.1366	12592
DLLPVAPEFPHPIPQF	45.19	1815.9559	16	2.2	908.98724	50.9804	22730
MVPVYE	45.17	736.3466	6	0.2	737.354	21.9076	9072
APSADAPM	45.12	758.3268	8	-1.9	759.3327	14.9479	5654
VDKPGPPIGPL	45.08	1088.6229	11	0.4	1089.63062	25.6334	10879

Peptide	-10LgP	Mass	Length	ppm	m/z	RT	Scan
KSYELPDGQVIT	45.06	1348.6874	12	4.4	1349.70068	26.5723	11349
SQFINFPIYL	45	1240.6492	10	2.5	1241.65955	53.9735	24013
ENGYQPQ	44.91	834.3508	7	1.5	835.35931	11.7875	4136
FWVHH	44.7	724.3445	5	1.5	725.35291	14.5656	5498
FDRVF	44.69	682.3438	5	-0.1	683.35107	21.5237	8861
DVHFE	44.68	645.2758	5	-1.4	646.28217	14.9479	5671
DLEFPGVV	44.67	874.4436	8	-1.7	875.4494	40.8104	18208
TPHVVLPPL	44.61	971.5804	9	-0.8	972.58685	31.7218	13844
GELFFWAH	44.59	1005.4708	8	-2.9	1006.47522	39.7794	17780
DLLPVAPEFPHPIPDFVL	44.49	2015.0768	18	1.2	1008.54681	54.3113	24165
HWH	44.48	478.2077	3	-1.1	479.21442	16.2299	6283
TEAPLN	44.42	643.3177	6	0.8	644.3255	12.8108	4623
TRDPSFFR	44.42	1024.509	8	-2.2	342.50952	19.7934	8075
GIDSIISF	44.41	850.4436	8	1.1	851.45184	41.2814	18482
FPGVVV	44.4	616.3584	6	-0.2	617.3656	31.074	13481
GLDFPNLPY	44.36	1034.5073	9	1.2	1035.51575	43.9309	19734
LPIDVITSPLY	44.36	1229.6907	11	3.2	1230.7019	46.3389	20838
DLEFPGVVVDGVA	44.27	1315.6659	13	3.7	1316.6781	44.916	20209
PSIVGRPR	44.27	880.5242	8	-1.8	441.26862	19.6673	7974
APEFPHPIPQFVLD	44.22	1605.8191	14	1	803.9176	40.5106	18091
DISADVK	44.2	746.381	7	-0.6	747.38782	11.8302	4155
KIWYH	44.13	745.3911	5	0.1	746.3985	13.623	5022
LLPVAPEFPHPIPDFVL	44.02	1900.0498	17	3	951.03497	53.5086	23817
AGPSDPSEA	43.94	829.3453	9	-1.4	830.3515	10.8539	3656

Peptide	-10LgP	Mass	Length	ppm	m/z	RT	Scan
DNVVPEVPE	43.93	996.4764	9	0.1	997.48376	26.4414	11314
HMDFPF	43.88	792.3265	6	-2.3	793.33197	33.0241	14473
HWFSLF	43.83	835.4017	6	1.1	836.40991	47.8162	21443
KIKIIAPPE	43.77	1007.6379	9	0.3	1008.64545	18.2533	7315
FISNPDE	43.74	820.3603	7	1.2	821.36853	16.9161	6632
APEFPHPIPD	43.72	1118.5397	10	0.6	1119.54761	26.3691	11265
KDLFAN	43.63	706.365	6	-0.4	707.37195	15.8888	6149
WLFF	43.62	611.3107	4	2.4	612.31952	50.8879	22792
SDYNIQ	43.59	738.3184	6	-2.2	739.3241	15.0773	5736
NQHGQVVKIF	43.57	1168.6353	10	2.5	1169.64551	20.009	8180
IHLPSPV	43.56	761.4435	7	0.1	762.45093	24.524	10446
KPH(+15.99)FGR	43.45	756.4031	6	-2.3	379.20795	1.0061	296
TKSDLEFPGVV	43.45	1190.6183	11	1.9	1191.62781	29.6535	12822
VASFIFPL	43.45	892.5058	8	1	893.51398	51.4567	22968
TVVTGPFPT	43.38	917.4858	9	2.5	918.49536	28.3979	12303
EAPLNPKANRE	43.37	1237.6415	11	-1	619.82739	10.9798	3740
RLPFIRPEDGGRY	43.36	1574.8317	13	0.2	525.95129	22.5923	9412
HHWFS	43.35	712.3081	5	-1.8	713.31415	14.9479	5653
FAPHTTY	43.34	835.3864	7	-0.1	836.39362	14.1357	5273
PGLSWVAVT	43.34	928.5018	9	0.5	929.50952	40.0784	17898
YLPTAPGEY	43.32	1009.4756	9	1.1	1010.48407	26.4013	11260
DIDESRWGA	43.23	1047.4622	9	-0.5	1048.46887	21.4369	8846
RDPSFF	43.21	767.3602	6	-2.8	384.68631	23.6244	10036
SIIENFLPS	43.19	1018.5335	9	1.9	1019.54272	40.4225	18083

Peptide	-10LgP	Mass	Length	ppm	m/z	RT	Scan
KGVNLPGVPVDLPA	43.18	1374.7871	14	5.3	1375.80164	36.0562	15970
FYNELR	43.13	840.413	6	0.8	421.21411	16.7381	6560
FINFPIYL	43.1	1025.5586	8	0.9	1026.56677	54.249	24142
SVHIKLPK	43.06	920.5807	8	0.8	921.58875	14.5662	5487
THQGPVIPR	43.03	1003.5563	9	1.7	1004.56531	12.3411	4410
GIITNWDDME	42.92	1192.5071	10	-0.4	1193.51379	39.2632	17474
LPVAPEFPHPI	42.92	1215.6652	11	7.5	1216.68152	33.8453	14895
YILDPIFK	42.88	1007.5692	8	2.1	1008.57855	35.758	15782
IIFF	42.84	538.3155	4	0.6	539.32312	41.878	18738
ILFF	42.84	538.3155	4	0.6	539.32312	41.878	18738
LIFF	42.84	538.3155	4	0.6	539.32312	41.878	18738
LLFF	42.84	538.3155	4	0.6	539.32312	41.878	18738
AVNDPFIAL	42.74	958.5123	9	-2.4	959.51733	40.8543	18277
SSVTVPDRL	42.72	972.524	9	-0.1	487.26923	21.8215	9056
DHLRII	42.69	765.4497	6	0	383.73212	18.8983	7622
DLLPVAPEFPHPIPQFVL	42.69	2028.1084	18	-0.4	1015.06104	54.4547	24238
TRDPSFF	42.65	868.4079	7	0.1	869.41528	22.5922	9376
KYLSKSIFDSLK	42.58	1427.8024	12	-0.7	714.90796	21.4785	8880
GIVLPPLYE	42.57	999.564	9	-0.9	1000.57043	41.0238	18416
GPPQAPQLQ	42.56	934.4872	9	-1.3	935.49323	18.6382	7445
WKDSYGYH	42.48	1054.4508	8	-0.1	1055.45801	13.7919	5111
GDRFLQ	42.45	734.3711	6	0.1	735.37848	15.4619	5905
TPLVPGDYYIA	42.44	1207.6124	11	9.5	1208.63123	37.0966	16473
FFW	42.4	498.2267	3	0	499.23398	38.9167	17290

Peptide	-10LgP	Mass	Length	ppm	m/z	RT	Scan
PVAILRSQQV	42.4	1109.6556	10	-6.3	1110.65601	42.0564	19061
DFVKPNLPF	42.3	1075.5702	9	-0.1	1076.57739	37.0942	16448
NLPVPVVGEPPATK	42.29	1416.7976	14	1.2	709.40692	27.5403	11815
QHHWFS	42.2	840.3667	6	-0.5	841.3736	15.2035	5773
APEFPHPIPQF	42.18	1278.6396	11	3.6	1279.65149	34.9679	15370
TLPWLQ	42.11	756.417	6	-3.7	757.42151	37.8542	16793
WWDGAK	42.05	761.3497	6	-0.9	762.35626	18.7243	7495
EDEDGFIPY	42.02	1083.4397	9	-1.3	1084.44556	34.3618	15124
YVAVIHSDLGHGIVLPPL	42.02	1899.0618	18	1.5	950.53961	40.5091	18047
GVAIDGELK	41.92	900.4916	9	2	901.50067	18.5584	7436
WLPALPF	41.92	842.4691	7	1.8	843.47778	52.6897	23370
DFTGTK	41.87	667.3177	6	-1.6	668.32391	10.6856	3563
FDPLIPAY	41.85	934.48	8	-1.7	935.48566	39.8649	17829
GHIYGPG	41.84	699.334	7	0.1	700.34137	13.3681	4918
RHPFDPPGAPGAPR	41.82	1470.7479	14	-0.7	491.25626	15.8037	6121
M(+42.01)DAIK	41.77	618.3047	5	0.5	619.31226	20.0328	8173
IYPAPQ	41.76	687.3591	6	0.6	688.36682	15.7606	6017
ADAPGPI	41.74	639.3228	7	1	640.33069	17.1791	6738
GELFFW	41.74	797.3748	6	2.4	798.38397	51.1898	22875
DPVLFPSFI	41.73	1033.5483	9	-1.1	1034.55457	52.3398	23296
IIAPPERKYSVW	41.72	1457.8031	12	-0.9	729.90814	25.6327	10873
QFTVGPL	41.7	760.4119	7	-1.9	761.41779	32.6378	14288
GISGIYLPK	41.6	946.5488	9	-0.9	947.55518	26.9305	11536
SSVTVPDRLSFE	41.6	1335.667	12	-0.4	668.84052	28.2305	12180

Peptide	-10LgP	Mass	Length	ppm	m/z	RT	Scan
LPDGQVITI	41.59	954.5386	9	2	955.54773	34.6208	15291
KISFPVL	41.58	802.4952	7	0.7	803.50311	33.4945	14660
MTQTPGKF	41.58	908.4426	8	3.3	909.45288	15.842	6124
WLPAPTW	41.53	869.4435	7	-2.4	870.44873	40.8952	18295
TRDPAFFR	41.52	1008.5141	8	-0.7	337.17841	20.698	8518
AVLSLYASGRTTGIVL	41.43	1619.9246	16	0.8	810.97021	38.6127	17143
DVALPGASKFF	41.36	1150.6022	11	2.5	1151.61243	37.7468	16774
RTLPWLQ	41.36	912.5181	7	-0.4	913.52502	29.4833	12840
TRDPAFF	41.35	852.413	7	-0.5	853.41986	23.4974	9848
VFFF	41.31	558.2842	4	0.8	559.29193	43.9731	19755
DLEFPGVVV	41.3	973.512	9	1.1	974.52039	45.1002	20370
TIGYKPT	41.29	778.4225	7	-0.7	390.21826	13.0244	4733
DFVFQPD	41.26	866.381	7	0.2	867.38843	33.5422	14744
GDDAPR	41.18	629.2769	6	0.8	630.28467	11.702	4096
SGPSIVH	41.18	695.3602	7	-2.5	696.36572	12.0429	4262
WFF	41.17	498.2267	3	-0.8	499.23355	42.6365	19138
AIPDPK	41.16	639.3591	6	-0.1	640.36633	11.2768	3862
SYFHPL	41.16	762.3701	6	1.3	763.3783	23.926	10051
HDFGYV	41.08	736.318	6	-1.1	737.32446	19.7095	8023
FVFQPD	41.07	751.3541	6	-0.2	752.36121	28.445	12297
GRIINVIGEPIDE	41.06	1423.7671	13	-0.2	712.89069	29.6149	12827
GRQGDPHGKF	41.05	1097.5366	10	-2.1	366.85205	7.9543	2359
VVPSPK	41.02	625.3799	6	-0.9	626.3866	9.4375	3024
VPVYE	40.95	605.306	5	-0.5	606.31305	16.358	6346

Peptide	-10LgP	Mass	Length	ppm	m/z	RT	Scan
TQTPGNF	40.85	763.35	7	0.7	764.35785	16.9551	6635
KFF	40.84	440.2423	3	0	441.24963	17.8239	7094
SDLLPVAPEF	40.82	1086.5597	10	4.1	1087.57141	43.6439	19625
HWFSL	40.78	688.3333	5	-1.4	689.3396	35.841	15856
RDPAFFR	40.76	907.4664	7	-0.7	454.74017	20.7842	8528
DDHFLF	40.75	792.3442	6	0.1	793.35162	34.0189	14935
AGDDAPRA	40.71	771.3511	8	-1.4	772.3573	4.2304	1204
YPGIAD	40.71	634.2962	6	-0.6	635.3031	18.8134	7597
PAPAAAP	40.62	593.3173	7	-0.8	594.3241	10.9798	3659
DVDGVAR	40.39	730.361	7	-0.7	731.36768	10.5162	3449
IKIIAPPERKY	40.39	1326.8024	11	-1.4	443.27411	17.5661	6950
HILLSLGE	40.38	880.5018	8	-0.1	881.50897	27.6267	11843
IDSFKLPAY	40.33	1052.5542	9	1.4	1053.56299	33.5422	14713
HHWFSL	40.23	825.3922	6	-1.8	826.39795	25.6779	10950
SVGDKIPADIRLVKIF	40.17	1770.0403	16	-0.5	886.02698	36.2718	16057
APLNPK	40.13	638.3751	6	-1.4	639.38153	8.2256	2399
NEHGIDIL	40.08	909.4556	8	1	910.46375	25.8111	10979
NEHGIDLL	40.08	909.4556	8	1	910.46375	25.8111	10979
CDIDIR	40.06	733.3428	6	-1.2	734.34924	19.6243	7960
RVPTPDVSVVDL	40.04	1295.7085	12	-2.1	648.86017	30.9875	13460
KFY	40.03	456.2373	3	0.2	457.24463	12.5997	4512
DRKGELFFW	39.99	1196.5978	9	0.3	599.30634	37.181	16491
PDGKF	39.93	562.2751	5	-0.6	563.28204	13.6655	5053
FEDVVGV	39.88	763.3752	7	-1.1	764.38159	28.4877	12255

Peptide	-10LgP	Mass	Length	ppm	m/z	RT	Scan
IAPPERKY	39.84	972.5392	8	-0.5	325.18686	12.2568	4337
GDDGVQDDL	39.82	932.3723	9	0.5	933.38007	20.3535	8338
LDKFYK	39.81	812.4432	6	1.7	813.45184	13.0244	4747
VVLPPLYEVTPH	39.77	1362.7546	12	0	682.38464	36.4012	16071
KYF	39.72	456.2373	3	0.2	457.24463	13.9629	5184
AVLSLYASGR	39.69	1035.5713	10	1.2	518.79352	23.2821	9749
FSLLIPVF	39.68	934.5527	8	-1.7	935.55847	55.0021	24515
QTPGNF	39.68	662.3024	6	0.4	663.30994	15.8037	6109
YWDHPIRD	39.62	1100.5039	8	1.1	1101.51245	16.5227	6430
KTFFD	39.6	656.317	5	-1.1	657.32349	21.735	9008
QIIPW	39.6	655.3693	5	0.6	656.37701	36.8753	16344
EPDDWLR	39.52	929.4243	7	0.5	930.43201	23.5393	9876
SKSIFDSL	39.51	895.4651	8	-0.1	896.47223	28.5311	12298
VHIKLPKLA	39.47	1017.6699	9	0.4	1018.67749	21.994	9090
DMPPGVM	39.43	745.3138	7	-1.3	746.32019	25.6779	10701
QTPGKF	39.43	676.3544	6	-0.5	339.18433	11.5317	3978
RKYSVW	39.4	837.4497	6	-2.4	419.73111	16.1866	6291
LIPVFE	39.37	716.4108	6	1.6	717.41925	35.5827	15672
PYYPY	39.33	701.3061	5	-0.1	702.31329	24.3958	10257
RTLPW	39.31	671.3755	5	0.4	672.38306	22.1666	9258
TFKLPGISPF	39.31	1105.6172	10	3.1	1106.62781	35.055	15491
VISSKWGEVR	39.3	1159.6349	10	-0.6	580.8244	17.6518	7014
ISPIYPE	39.29	817.4221	7	0.7	818.42999	24.8095	10510
DSGVGIY	39.26	709.3282	7	0	710.33551	22.1232	9176

Peptide	-10LgP	Mass	Length	ppm	m/z	RT	Scan
AQSKRGILTLKYPIE	39.23	1715.9934	15	0.1	859.00403	23.4146	9810
AKDPWDEPGKIL	39.22	1367.7085	12	3.4	1368.72034	27.5836	11849
FDPIIE	39.22	732.3694	6	-2.3	733.37494	29.9576	12966
YDFGFV	39.22	746.3275	6	0.6	747.33527	39.1748	17453
DKFYK	39.19	699.3591	5	-0.9	700.36578	9.5881	3027
AIYPAPQ	39.18	758.3962	7	1.6	759.40472	18.5571	7416
ELPDGQ	39.16	657.2969	6	-0.7	658.30377	12.7264	4533
FPHPIPDF	39.15	968.4756	8	-2.5	969.48041	32.7237	14268
FWWDGAK	39.13	908.418	7	-0.4	909.42493	30.4208	13219
TFNTPA	39.11	649.3071	6	-1.2	650.3136	15.3314	5857
VITIGNE	39.08	744.4017	7	1.4	745.4101	20.0523	8187
TDAIPDPK	39.06	855.4338	8	-2.2	428.72324	13.7074	5065
MYVAIQ	39.05	723.3625	6	4.2	724.37286	23.7536	9982
EDVDGVA	39	703.3024	7	-0.7	704.3092	11.9029	4217
GYSFTT	39	674.2911	6	0.8	675.29895	19.2823	7788
IPVFE	38.94	603.3268	5	-1	604.33344	26.9183	11513
GIIEYPF	38.88	837.4272	7	0.7	838.43506	40.4225	18086
WLDVIIT	38.87	858.4851	7	0.7	859.49292	43.2361	19450
HWFSLFN	38.86	949.4446	7	-1.2	950.45074	41.4935	18569
IIAPPERKYSVWIG	38.86	1627.9086	14	-0.2	814.96143	28.6172	12339
YELPDGQVI	38.84	1032.5127	9	-2.7	1033.51721	32.7237	14334
HRFVKPF	38.83	929.5236	7	-0.3	310.84836	14.6541	5534
VPIYE	38.82	619.3217	5	1.1	620.32965	19.3254	7787
MYPGIAD	38.78	765.3367	7	0.2	766.34412	22.7637	9471

Peptide	-10LgP	Mass	Length	ppm	m/z	RT	Scan
KSYELPDGQVITI	38.76	1461.7715	13	1.2	731.89392	35.3588	15584
TRDPAFFRL	38.74	1121.5981	9	-0.8	374.87302	29.3973	12727
ESVPILK	38.73	784.4694	7	0.1	785.47681	17.3508	6852
FNVWTR	38.71	821.4184	6	1.4	411.71704	19.9234	8127
EEPIQHPQ	38.69	976.4614	8	0.6	977.4693	11.4882	3973
GILTLKYPI	38.68	1016.627	9	1.6	1017.63586	36.0995	15963
EHPVLL	38.65	706.4014	6	1.5	707.40967	20.0523	8166
IPIFAQVK	38.57	914.5589	8	3.5	915.5694	27.8857	11991
WDHPIRDGF	38.55	1141.5305	9	0.3	1142.53809	21.264	8758
WFSLFN	38.55	812.3857	6	0.3	813.39319	47.3875	21286
HKYMDNIFK	38.53	1194.5856	9	-0.2	598.29993	17.3938	6872
KIWYHTF	38.5	993.5072	7	-1.2	994.51331	21.9719	9121
GEFVY	38.47	613.2748	5	0	614.28204	24.0074	10083
SPPVILNEFLGPA	38.47	1352.734	13	2.4	1353.74451	46.2548	20776
DGFIPY	38.46	710.3275	6	-0.5	711.33447	38.2	17024
RGYSFTTT	38.44	931.4399	8	-0.7	466.7269	15.2053	5799
VLDQIAF	38.39	804.4381	7	0.7	805.44598	35.2287	15562
SLLIPVFE	38.38	916.5269	8	2	917.53607	48.2875	21631
VVDGNKIV	38.38	842.4861	8	0.9	843.4942	15.5056	5946
AKNPYDVPTAPGRPE	38.36	1610.8052	15	1	806.41071	17.9966	7168
LDPIFK	38.31	731.4218	6	-1.4	732.42798	26.9183	11479
SDLLPVAPEFPHPIPDFVLEQ	38.31	2359.21	21	1	1180.6134	52.9198	23519
GMDFW	38.25	654.2472	5	1.1	655.25519	37.572	16656
MDFWL	38.24	710.3098	5	1.5	711.31812	47.3747	21269

Peptide	-10LgP	Mass	Length	ppm	m/z	RT	Scan
IFF	38.16	425.2314	3	-0.3	426.23859	32.1638	14047
LFF	38.16	425.2314	3	-0.3	426.23859	32.1638	14047
RDPSFFRL	38.15	1036.5454	8	1.6	519.28082	29.2237	12672
GFAGDDAPRA	38.12	975.441	10	0.8	488.72815	14.5662	5502
KISFPVLL	38.12	915.5793	8	-1.5	916.58521	40.0784	17873
SDNVADVD	38.1	833.3403	8	0	834.34753	13.4101	4923
WITKEEYDE	38.09	1211.5347	9	0.6	1212.5426	19.8386	8084
IHDADEA	38.05	769.3242	7	-1	770.33069	9.9691	3205
SIFDSLK	38.03	808.433	7	-0.9	809.43964	23.6676	9933
FSLLIPVFE	37.96	1063.5953	9	2.7	1064.60547	53.7313	23894
GDPHGKF	37.96	756.3555	7	-1.9	379.1843	5.0121	1466
DKSGNHIDIR	37.9	1153.584	10	-0.9	385.53491	10.8957	3696
HDDKVTVR	37.89	968.5039	8	-0.6	323.84174	0.7726	43
YSNVKIT	37.89	823.444	7	0.1	824.45135	14.4798	5423
IEPITDPNLR	37.85	1166.6295	10	1.9	584.32312	22.0367	9164
DENRLL	37.83	758.3923	6	-0.2	759.39935	17.3111	6820
KVSSTL	37.81	633.3698	6	-0.3	634.37683	10.18	3318
SPW	37.81	388.1747	3	-1.2	389.18149	17.3938	6883
MVPFPR	37.79	745.3945	6	0	746.40179	20.7844	8480
LPVAPE	37.78	624.3483	6	0.3	625.35571	17.9966	7119
GGEFPARPDNKE	37.68	1315.6156	12	0.5	658.81543	13.1963	4767
LPPITLPEPS	37.65	1062.5961	10	2.4	1063.60596	35.7146	15763
AAPGEKPF	37.63	815.4177	8	1.9	816.42651	14.6529	5514
IKVSYF	37.62	755.4218	6	1.9	756.43048	26.5298	11303

Peptide	-10LgP	Mass	Length	ppm	m/z	RT	Scan
VGDKIPA	37.62	698.3962	7	0	699.4035	12.7259	4607
NIIIVPGY	37.6	887.5117	8	0.3	888.51917	36.5728	16174
SLFQPS	37.57	677.3384	6	1.6	678.3468	23.1957	9692
DLGLPFTIH	37.56	1011.5389	9	-3.6	1012.54254	40.1193	17924
TERGYSFTTT	37.5	1161.5302	10	0.5	581.77264	15.8442	6113
APAPY	37.45	517.2536	5	0.2	518.26099	13.4101	4905
WLDVVDE	37.45	874.4072	7	-3.7	875.41125	34.1912	15043
EGPGSPEEPT	37.44	998.4193	10	1.1	999.42761	12.939	4710
YKPTEPGYY	37.44	1116.5128	9	0.8	1117.52087	18.2102	7265
GPLGSDEYV	37.4	935.4236	9	0.3	936.43115	22.0818	9142
TLKYPIE	37.4	862.48	7	0.9	863.48804	20.3093	8299
RDPAFF	37.37	751.3653	6	-1.9	752.37115	23.5814	9869
IPW	37.36	414.2267	3	-0.7	415.23367	27.7162	11897
LPW	37.36	414.2267	3	-0.7	415.23367	27.7162	11897
DLEFPGV	37.35	775.3752	7	-1.4	776.38135	38.0269	16858
VTPHLF	37.33	712.3908	6	-0.6	713.39764	19.6243	7957
TESSVTVPDRLSFE	37.3	1565.7573	14	-1.9	783.88446	28.9624	12520
YIDGDVK	37.25	808.3967	7	-1.1	809.40308	12.0646	4272
AVFPSIV	37.22	731.4218	7	-0.8	732.42841	36.3583	16150
SVVPSPK	37.17	712.4119	7	1.1	713.41998	12.428	4370
TPGNF	37.17	534.2438	5	-0.3	535.25092	15.12	5713
FAGDDAPRA	37.1	918.4195	9	1.4	919.4281	13.1963	4814
SHPAPM	37.09	638.2846	6	-0.5	639.29156	9.3888	2940
FQPSFL	37.06	737.3748	6	-1.8	738.38074	32.6804	14364

Peptide	-10LgP	Mass	Length	ppm	m/z	RT	Scan
ELPPPFHHQ	37.04	1100.5403	9	-0.5	1101.547	21.5211	8688
